# The mevalonate pathway contributes to breast primary tumorigenesis and lung metastasis

**DOI:** 10.1002/1878-0261.13716

**Published:** 2024-08-09

**Authors:** Javier Conde, Isabel Fernández‐Pisonero, L. Francisco Lorenzo‐Martín, Rocío García‐Gómez, Berta Casar, Piero Crespo, Xosé R. Bustelo

**Affiliations:** ^1^ Molecular Mechanisms of Cancer Program, Centro de Investigación del Cáncer Consejo Superior de Investigaciones Científicas (CSIC) and Universidad de Salamanca Spain; ^2^ Instituto de Biología Molecular y Celular del Cáncer CSIC and Universidad de Salamanca Spain; ^3^ Centro de Investigación Biomédica en Red de Cáncer (CIBERONC) Instituto de Salud Carlos III Madrid Spain; ^4^ Instituto de Biomedicina y Biotecnología de Cantabria (IBBTEC) CSIC and Universidad de Cantabria Santander Spain; ^5^ Present address: Molecular and Cellular Gastroenterology Group Instituto de Investigación Sanitaria Santiago de Compostela Santiago de Compostela Spain; ^6^ Present address: Laboratory of Stem Cell Bioengineering École Polytechnique Fédérale de Lausanne Lausanne Switzerland

**Keywords:** cholesterol, guanosine nucleotide exchange factors, oncogenic signaling, RAC1, RHO GTPases, sterol regulatory element‐binding protein

## Abstract

The mevalonate pathway plays an important role in breast cancer and other tumor types. However, many issues remain obscure as yet regarding its mechanism of regulation and action. In the present study, we report that the expression of mevalonate pathway enzymes is mediated by the RHO guanosine nucleotide exchange factors VAV2 and VAV3 in a RAC1‐ and sterol regulatory element‐binding factor (SREBF)‐dependent manner in breast cancer cells. Furthermore, *in vivo* tumorigenesis experiments indicated that the two most upstream steps of this metabolic pathway [3‐hydroxy‐3‐methylglutaryl‐coenzyme A synthase 1 (HMGCS1) and 3‐hydroxy‐3‐methylglutaryl‐coenzyme A reductase (HMGCR)] are important for primary tumorigenesis, angiogenesis, and cell survival in breast cancer cells. HMGCR, but not HMGCS1, is also important for the extravasation and subsequent fitness of breast cancer cells in the lung parenchyma. Genome‐wide expression analyses revealed that HMGCR influences the expression of gene signatures linked to proliferation, metabolism, and immune responses. The HMGCR‐regulated gene signature predicts long‐term tumor recurrence but not metastasis in cohorts of nonsegregated and chemotherapy‐resistant breast cancer patients. These results reveal a hitherto unknown, VAV‐catalysis‐dependent mechanism involved in the regulation of the mevalonate pathway in breast cancer cells. They also identify specific mevalonate‐pathway‐dependent processes that contribute to the malignant features of breast cancer cells.

## Graphical abstract

In this work, Conde *et al.* describe a new VAV1–RAC1–SRBEF‐dependent pathway that promotes the expression of the mevalonate pathway in breast cancer cells. They also show that key enzymes of this metabolic route (HMGCS1 and HMGCR) play roles in breast primary tumorigenesis and in specific steps of the metastatic dissemination of the cancer cells to the lung parenchyma.

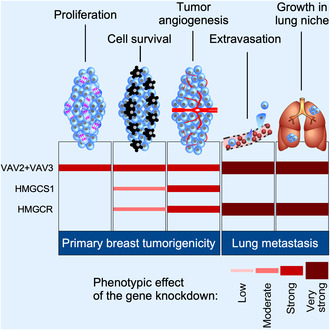

**Abbreviations**
a.u.: arbitrary units
ABC: ATP‐binding cassette
AMPK: 5′ AMP‐activated protein kinase
DMEM: Dulbecco's modified Eagle medium
GDP: guanosine diphosphate
GEF: guanosine nucleotide exchange factor
GSEA: gene set expression analyses
GTP: guanosine triphosphate
HIF1α: hypoxia‐inducible factor 1α
HMGCR: 3‐hydroxy‐3‐methylglutaryl‐coenzyme A reductase
HMGCS1: 3‐hydroxy‐3‐methylglutaryl‐coenzyme A synthase 1
MEK: mitogen‐activated protein kinase
mTORC1: mammalian target of rapamycin complex 1
MTT: 3‐(4,5‐dimethylthiazol‐2‐yl)‐2,5‐diphenyl‐2H‐tetrazolium bromide
p140^CAS^
: p130 Crk‐associated substrate‐associated protein of 140 kDa
PAK: p21‐activated kinases
PI3K: phosphatidyl inositol 3‐kinase
qRT–PCR: quantitative reverse transcription polymerase chain reaction
SEM: standard error of the mean
SREBF: sterol regulatory element‐binding factor

## 1 Introduction

Metabolic rewiring is required to provide a supply of both energy and biosynthetic products to maintain the aberrant proliferative and metabolic demands of cancer cells. In addition, it can facilitate the modulation of specific signaling pathways and genetic programs [1, 2]. This reprogramming can result from direct genetic alterations (mutations, copy number changes) in metabolic genes and/or from the emergence of mutations in signaling proteins and transcription factors that regulate the expression and/or activity of enzymes involved in the main metabolic routes [2]. One of the metabolic routes that is found consistently upregulated in cancer cells is the mevalonate pathway, which favors the biosynthesis of sterols, isoprenoids, and cholesterol in cancer cells [3, 4]. In the last decade, this metabolic route has gained much attention for its contribution to cell transformation and tumorigenesis in several types of cancer, including breast cancer [3, 5]. Consistent with this, ectopic expression of specific enzymes of this route increases colony formation in breast cancer cells [6]. Likewise, it has been reported that both cholesterol and 27‐hydroxycholesterol promote breast tumorigenesis in preclinical models by promoting angiogenesis and an immunosuppressive microenvironment [7, 8]. The analysis of patient data revealed that high levels of expression of HMGCR, the rate‐limiting enzyme of the mevalonate pathway [3, 5], are associated with increased proliferation of breast tumors [9]. These observations led to test the relevance of HMGCR inhibitors (i.e., statins) as antiproliferative drugs for treating breast cancer. However, it is worth noting that the studies addressing the effects of both statins and cholesterol levels have yielded rather contradictory results up to now in this tumor type in the clinical setting [5, 10, 11, 12].

The expression of mevalonate pathway‐related genes can be promoted in cancer cells via the stimulation of the phosphatidyl inositol 3‐kinase α (PI3K)–AKT–mammalian target of rapamycin complex 1 (mTORC1) pathway, TP53 and TP53 mutants, retinoblastoma, MYC, and E2F family proteins [3, 4]. An essential hub of this framework is the family of SREBFs, a group of transcription factors that promote the transcription of all mevalonate pathway‐related genes either *per se* or in cooperation with other transcription factors such as MYC and mutant versions of TP53 [4, 13]. The activity of the mevalonate pathway can be further enhanced via the supply of key metabolic components from other metabolic pathways, such as glycolysis, oxidative phosphorylation, and glutamine or acetate catabolic consumption [3, 4].

The VAV family comprises three RHO guanosine nucleotide exchange factors (GEFs) that catalyze the transition of RAC1 and to a lesser extent other RHO GTPases from the inactive (GDP‐bound) to the active (GTP‐bound) state [14, 15]. In addition to this main biochemical function, VAV proteins can also play noncatalytic, adaptor‐like functions in cells [14, 15, 16, 17]. In mammals, the VAV family is composed of three members: VAV1, VAV2, and VAV3 [14, 15]. VAV1 is predominantly expressed in hematopoietic cells, whereas VAV2 and VAV3 exhibit more ubiquitous expression patterns [18, 19, 20, 21]. In addition to playing critical roles in the hematopoietic, nervous, and cardiovascular systems [14, 15, 16, 17, 22], these proteins have either protumorigenic (catalysis‐dependent) or tumor suppressor (catalysis‐independent) properties in a variety of cancer cell types [14, 15, 16, 17]. In particular, VAV1 has been shown to have tumor suppressor functions in TLX^+^ T cell receptor‐negative T cell acute lymphoblastic leukemia and both protumorigenic and tumor suppressor roles in peripheral T cell lymphoma [16, 23]. These activities can be deregulated by mutations and/or the spurious alteration of upstream regulators [16, 23]. However, to date, the role of VAV2 and VAV3 in tumors has been limited to the deregulation of endogenous wild‐type proteins [24, 25, 26]. This is the case for breast cancer, where the endogenous wild‐type versions of VAV2 and VAV3 regulate signaling and transcriptional programs that favor primary tumorigenesis and several lung metastasis dissemination stages, such as intravasation, extravasation, and fitness in the lung parenchyma after the extravasation step [27, 28]. Several distal elements of these protumorigenic and metastatic programs have been identified [27, 28].

To further dissect these programs, we performed further *in silico* analyses of the previously described VAV‐dependent transcriptome of the breast cancer cell line 4T1 [27, 28] to identify additional proteins and biological programs that could be associated with the proliferative and malignant properties of breast cancer cells. The 4T1 cell line [29] is one of the most widely used breast cancer models for several reasons: (a) it has molecular features similar to those of triple‐negative breast cancer models (estrogen receptor‐negative, progesterone receptor‐negative, epidermal growth factor receptor 2‐negative); (b) it allows the use of immunocompetent recipient mice to assess *in vivo* tumorigenic and metastatic properties using syngeneic models; (c) it has nonmetastatic counterparts to facilitate comparative studies; and (d) it has been recently comprehensively characterized at the genomic level [29, 30]. Our bioinformatic analyses revealed that the expression of the enzymes in the entire mevalonate pathway is VAV‐dependent in 4T1 cells. In this work, we provide wet‐lab data supporting the VAV‐, RAC1‐, and SREBF‐dependent regulation of the mevalonate pathway in 4T1 cells. Moreover, we demonstrate that two key regulatory components of this pathway, HMGCS1 and the rate‐limiting enzyme HMGCR, play specific roles in primary tumorigenesis and lung metastasis.

## 2 Materials and methods

### 2.1 Ethics statement

All animal experiments carried out in this study were performed in accordance with the study protocol approved by the Bioethics Committee of Salamanca University and the animal experimentation authorities of the autonomous government of Castilla y León (Spain) (animal license numbers #315 and #568). Mice were kept in ventilated rooms in pathogen‐free facilities under controlled temperature (23 °C), humidity (50%), and illumination (12 h light/12 h dark cycle) conditions. After weaning, mice were fed a standard chow global diet #2918 (6.2% fat, 3.1 kcal·g^−1^; Harlan Laboratories, Indianapolis, IN, USA). Animals were treated humanely in accordance with standards described in the Guide for the Care and Use of Laboratory Animals, considering relevant national and European guidelines. The part of our study involving animal work is reported in accordance with the ARRIVE guidelines. No human samples were used in this study.

### 2.2 Cell culture

All cells used in this study were incubated at 37 °C with 5% CO_2_. 4T1 cells (RRID: CVCL_0125) (CRL–2539™; ATCC, Manassas, VA, USA) [29] and their derivatives (KD_2_, KD_3_, KD_2/3_, KD_2/3_ + V2 + V3; KD_2/3_ + RAC1^F28L^) were cultured in Roswell Park Memorial Institute (RPMI) media (Cat. No. R8758; Sigma‐Aldrich, Saint Louis, MO, USA) supplemented with 10% fetal bovine serum (Cat. No. 10500064; ThermoFisher Scientific, Waltham, MA, USA) and penicillin (100 U·mL^−1^)‐streptomycin (100 μg·mL^−1^) (Cat. No. 15140‐122; Thermo Fisher Scientific). The nonmetastatic 67NR (RRID: CVCL_0125), 4OT7 (RRID: CVCL_B383), and 168FARN (RRID: CVCL_0I86) cell lines were kindly provided by F. Miller (Wayne State University School of Medicine, Detroit, MI, USA) [29] and maintained in culture as described above. The Lenti‐X 293T lentiviral packaging cell line (RRID: CVCL_4401) (Cat. No. 632180; Clontech, Mountain View, CA, USA) was cultured in Dulbecco's modified Eagle medium (DMEM) (Cat. No. D0819; Sigma‐Aldrich) supplemented with 10% tetracycline‐free fetal calf serum, penicillin (100 U·mL^−1^)‐streptomycin (100 μg·mL^−1^). Cell lines were authenticated before performing the study at the Salamanca University Clinical Hospital using the short tandem repeat‐based method. Mycoplasma‐free status of cell lines was confirmed at our own lab using the HEK‐Blue Selection kit (Cat. No. hb‐sel; InvivoGen, San Diego, CA, USA). When required, cells were stimulated with insulin growth factor 1 for the indicated periods of time. To this end, 4T1 cells were maintained in overnight serum‐starved conditions and, subsequently, stimulated with 50 ng·mL^−1^ of insulin growth factor 1 (Cat. No. 100‐11; Peprotech, Cranbury, NJ, USA) for the indicated periods of time. Cells were then washed three times with ice‐cold phosphate‐buffered saline solution and lysed as indicated below (see Section 2.13).

### 2.3 Microbe strains

The *Escherichia coli* DH5α strain was used for cloning and plasmid generation.

### 2.4 Plasmid construction

The lentiviral vectors encoding HA‐tagged wild‐type VAV2 (pCCM33), MYC‐tagged wild‐type VAV3 (pCCM31), and HA‐tagged RAC1^F28L^ (pMMM1) have been described previously [27]. In addition to exhibiting antibiotic resistance, the pCCM31 plasmid bicistronically encodes a green fluorescent protein. Mammalian expression vectors encoding mutant versions of GFP‐tagged RAC1 (Q61L) (pNM42), RHOA (Q63L) (pNM041), RHOG (Q61L) (pVOS17), or CDC42 (Q61L) (pNM040) have been described previously [31, 32, 33, 34]. Plasmids encoding untagged wild‐type RAC1 (pCEFL‐AU5‐RAC^WT^), RAC1^Q61L^ (pCEFL‐AU5‐RAC)1(QL), RAC1^G15A^ (pJCA04), and RAC1^T17N^ (pACC12) have been described elsewhere [31, 32, 33, 34, 35]. The pRL‐SV40 vector (encoding the *Renilla* luciferase) was obtained from Promega (Madison, WI, USA; Cat. No. E2231). The pSynSRE‐T‐Luc vector (reporter plasmid encoding luciferase under the control of the *Hmgcs1* promoter) was obtained from Addgene (Watertown, MA, USA; Cat. No. 60444). To generate the lentivirus encoding HMGCR (pLVX‐Hmgcr), we used the GeneArt Gene Synthesis service (Thermo Fisher Scientific). Briefly, the full‐length sequence of mouse HMGCR obtained from synthetic oligonucleotides and/or PCR products was inserted into the pLVX‐IRES‐Hyg vector (Cat. No 632185; Clontech) using *NotI* and *SpeI* cloning sites. All vectors were verified by DNA sequencing at the CIC Genomics Facility.

### 2.5 Lentiviral particle production

We transfected Lenti‐X 293T cells with the indicated lentiviral vectors using the Lenti‐X HT packaging mix (Cat. No. 631248; Clontech). Viral particles were collected 48 h after the transfection step and concentrated using a Lenti‐X Concentrator Kit (Cat. No. 631232; Clontech). Viral titers were quantified using the Lenti‐X qRT–PCR Titration Kit following the manufacturer's instructions (Cat. No. 631235; Clontech).

### 2.6 Generation of knockdown cell lines

4T1 cells with depleted *Vav2* (KD_2_), *Vav3* (KD_3_), or *Vav2* plus *Vav3* (KD_2/3_) transcripts were generated, validated, and characterized in a previous study [27]. Briefly, the parental cells were transduced with prepackaged Mission TRC lentiviral particles encoding shRNAs targeting the mouse *Vav2* mRNA (Cat. No. TRC0000097094; Sigma‐Aldrich) and the mouse *Vav3* mRNA (Cat. No. TRC0000097124; Sigma‐Aldrich). As a control, we used 4T1 cells that were transduced with lentiviruses lacking shRNAs (pLKO). In all cases, the cells were incubated with lentiviral particles in the presence of polybrene (8 μg·mL^−1^) (Cat. No. H9268; Sigma‐Aldrich) and selected with puromycin (2 μg·mL^−1^) (Cat. No. P8833; Sigma‐Aldrich). We followed a similar approach to generate the *Hmgcs1* or *Hmgcr* knockdown 4T1 cell clones using lentiviral particles encoding shRNAs targeting each of these transcripts (Cat. No TRC0000076043 and TRC0000173307, respectively; Sigma‐Aldrich).

### 2.7 Ectopic expression of proteins in 4T1 knockdown cell lines

The generation, validation, and characterization of KD_2/3_ cells overexpressing either VAV2 plus VAV3 (KD_2/3_ + V2 + V3) or RAC1^F28L^ (KD_2/3_ + RAC1^F28L^) have been reported elsewhere [27, 28, 36]. To generate KD_2/3_ + V2 + V3 cells, we infected the KD_2/3_ cell line stepwise with lentiviral particles encoding HA‐tagged VAV2 (pCCM33) and MYC‐tagged VAV3 (pCCM31) as described above and, subsequently, selected them using 200 μg·mL^−1^ hygromycin (Cat. No. 10843555001; Sigma‐Aldrich) (for VAV2‐positive cells) and flow cytometry (to select, based on the bicistronic expression of the GFP, the VAV3‐positive cells). To generate HMGCR‐overexpressing 4T1 cells, cells were infected with lentiviral particles encoding HMGCR (pLVX‐Hmgcr) in the presence of polybrene and subsequently selected with hygromycin as indicated above.

### 2.8 Detection of the expression of mevalonate pathway genes in breast cancer cells

Heatmaps for all transcripts encoding direct mevalonate pathway components were generated using the heatmap3 package [36] and the GEO datasets GSE33348 [27] and GSE160101 [37].

### 2.9 Gene set expression analyses

They were performed with the described gene sets using gene set permutations (*n* = 1000) for the assessment of significance and signal‐to‐noise metrics for ranking genes [38]. The signature for SREBF targets was obtained from a previous report [39].

### 2.10 Determination of mRNA abundance

Total RNA was extracted from the indicated cells using NZYol (Cat. No. MB18501; NZYtech, Lisboa, Portugal), and RT–PCR was performed using the Power SYBR Green RNA‐to‐C_T_ 1 Step Kit (Cat. No. 4389986; Applied Biosystems, Foster City, MA, USA). The raw data were subsequently analyzed with the stepone software v2.1 (Applied Biosystems). Primers used were (in alphabetical order): 5′‐GCA ATC CTG GGC CCC ACA TTC ACT‐3′ (*Hmgcr*, forward); 5′‐GCA GCA CAT GAT CTC CAG CTG GCG‐3′ (*Hmgcr*, reverse); 5′‐CCC CTT CAC AAA TGA CCA CA‐3′ (*Hmgcs1*, forward); 5′‐GAC AGC TGA TTC AGA TTC G‐3′ (*Hmgcs1*, reverse); 5′‐GCG AGC TGG AAG AGA GAA CAA‐3′ (*Idi1*, forward); 5′‐AGG GTA TTC CCA ACT CGG CT‐3′ (*Idi1*, reverse); 5′‐GCC TCA GGA CCT AAT GGT C‐3′ (*Mvd*, forward); 5′‐AGT TGA TGG GCA GGA TCA GC‐3′ (*Mvd*, reverse); 5′‐CAG CTC AGA GCC GTG GTG A‐3′ (*Srebp1*, forward); 5′‐TTG ATA GAA GAC CGG TAG CGC‐3′ (*Srebp1*, reverse); 5′‐CAC AAT ATC ATT GAA AAG CGC TAC CCG TTC‐3′ (*Srebp2*, forward); and 5′‐TTT TTC TGA TTG GCC AGC TTC AGC ACC ATG‐3′ (*Srebp2*, reverse). For normalization control, we used the abundance of the endogenous *Gapdh* transcript using primers 5′‐TGC ACC ACC AAC TGC TTA GC‐3′ (forward) and 5′‐TCT TCT GGG TGG CAG TGA TG‐3′ (reverse). When appropriate, cells were treated with the indicated concentrations of rapamycin (Sirolimus, Cat. No. S1039; Selleckchem, Houston, TX, USA), torin1 (Cat. No. S2827; Selleckchem), torkinib (PP242, Cat. No. S2218; Selleckchem), and LY294002 (2.5 μm; SF1101, Cat. No. S1105; Selleckchem) for 24 h prior to RNA extraction and qRT–PCR determinations.

### 2.11 Luciferase reporter assays

To measure the stimulation of SREBP activity in control, KD_2/3_ and KD_2/3_ + V2 + V3 cells, the cells were cotransfected with pRL‐SV40 (5 ng) plus the pSynSRE‐T‐Luc (1 μg) vectors. In the case of transient transfections, the parental 4T1 cells were transfected using liposomes with pRL‐SV40 (5 ng), pSynSRE‐T‐Luc (1 μg), and the indicated testing plasmids. When needed, the cells were supplemented with empty vectors to maintain a constant the total amount of transfected DNA among the samples. In all cases, cells were lysed 48 h posttransfection with Passive Lysis Buffer (5×), and luciferase activities were determined using the Dual Luciferase Assay System (Cat. No. E1910; Promega). In all cases, the firefly luciferase activity values obtained at each experimental point were normalized considering the activity of the *Renilla* luciferase obtained from the same sample. In addition, we analyzed aliquots of the same lysates by Western blotting to assess the expression of the ectopically expressed proteins in the appropriate experimental sample. The values are represented in the figures as the *n*‐fold change of the experimental sample relative to the SREBP activity shown by control cells (which was given an arbitrary value of 1 in each case). When indicated, cells were treated with the inhibitors rapamycin (2.5 μm), torin1 (2.5 μm), torkinib (2.5 μm), LY294002 (10 μm; Cat. No. S1105; Selleckchem), wortmannin (200 nm; Cat. No. S2758; Selleckchem), PIK‐75 (100 nm; Cat. No. S1205; Selleckchem), or U0126 (25 μm; Cat. No. S1102; Selleckchem).

### 2.12 Immunofluorescence analyses

Indicated 4T1 cells were cultured onto coverslips treated with 2 μg·mL^−1^ fibronectin in imaging buffer (20 mm HEPES [pH 7.4], 140 mm NaCl, 2.5 mm KCl, 1.8 mm CaCl_2_ and 1 mm MgCl_2_) for 1 h at 37 °C. Cells were seeded and allowed to attach for 24 h and, subsequently, fixed in 4% formaldehyde in phosphate‐buffered saline solution for 10 min at room temperature. After washing twice with phosphate‐buffered saline solution, cells were permeabilized with 0.3% Triton X‐100 (Cat. No. X100; Sigma‐Aldrich) in phosphate‐buffered saline solution for 10 min at room temperature, washed three times with phosphate‐buffered saline solution, and blocked in 2% bovine serum albumin (Cat. No. MB04603; NZYtech) in TBS‐T for 30 to 45 min in a wet chamber. When indicated, cells were incubated for 2 h at room temperature with antibodies to SREBF1 (Cat. No. sc‐13551; Santa Cruz Biotechnology, Dallas, TX, USA) or SREBF2 (Cat. No. ab30682; Abcam, Cambridge, UK), washed thrice in TBS‐T, incubated with the corresponded secondary antibody [goat antibodies to mouse IgG coupled to Alexa Fluor 488 (Cat. No. A32723, 1 : 400 dilution; Invitrogen, Carlslbad, CA, USA) or goat anti‐rabbit IgG coupled to Alexa Fluor™ 488 (Cat. No. A‐11034; Thermo Fisher Scientific)] for 30 min at room temperature. After washing twice with TBS‐T, cells were finally stained with 1 μg·mL^−1^ of 4′,6‐diamidino‐2‐phenylindole (Cat. No. D1306; Invitrogen) for 3 min to visualize the nuclei. Coverlips were mounted on microscopy slides with Mowiol medium (Cat. No. 475904; Merck, Rahway, NJ, USA) for microscopy analyses using a Laser Scan Leica SP8 Confocal Microscopy (Wetzlar, Germany). Subcellular distribution of SREBF was done *de visu*, counting in each experiment a total of at least 100 cells/slide.

### 2.13 Immunoblotting

The indicated cultured cells were washed with phosphate‐buffered saline solution and lysed in 10 mm Tris–HCl (pH 8.0), 150 mm NaCl, 1% Triton X‐100 (Cat. No. 93443; Sigma‐Aldrich), 1 mm Na_3_VO_4_ (Cat. No. S6508; Sigma‐Aldrich), 10 mm β‐glycerophosphate (Cat. No. G9422; Sigma‐Aldrich), and a mixture of protease inhibitors (Cat. No. 11836170001; Roche, Basel, Switzerland). Cellular extracts were precleaned by centrifugation at 12 000 ***g*** for 10 min at 4 °C, denatured by boiling in 4× SDS/PAGE sample buffer, separated electrophoretically, and transferred onto nitrocellulose filters using the iBlot Dry Blotting System (Cat. No. IB21001; ThermoFisher Scientific). The membranes were blocked in 5% bovine serum albumin (Cat. No. A7906; Sigma‐Aldrich) in TBS‐T (25 mm Tris–HCl (pH 8.0), 150 mm NaCl, 0.1% Tween‐20) for at least 1 h and then incubated overnight with the appropriate antibodies. The membranes were then washed three times with TBS‐T, incubated with the appropriate secondary antibody (1 : 2000 dilution) (Cat. No. GENA93401M; Cytiva, Malborough, MA, USA) for 60 min at room temperature and washed three times as described above. Immunoreacting bands were visualized using the chemiluminescent method ECL Pierce (Cat. No. 32106; ThermoFisher Scientific). The primary antibodies used included those to EGFP (1 : 2000 dilution; Cat. No. MMS‐118P; Covance, Princeton, NJ, USA), RAC1 (1 : 2000 dilution; Cat. No. 610651; BD Biosciences, Franklin Lakes, NJ, USA), phospho‐AKT (1 : 1000 dilution, p‐Thr^308^; Cat. No. 4056; Cell Signaling Technology, Danvers, MA, USA), phospho‐AKT (1 : 1000 dilution, p‐Ser^473^; Cat. No. 4051; Cell Signaling Technology), total AKT (1 : 1000 dilution; Cat. No. 2920; Cell Signaling Technology), phospho‐ERK1/2 (1 : 1000 dilution, p‐Thr^202^ and p‐Tyr^204^; Cat. No. 4370; Cell Signaling Technology), total ERK (1 : 1000 dilution; Cat. No. 9102; Cell Signaling Technology), phospho‐p70 S6K (1 : 1000 dilution, p‐Thr^389^; Cat. No. 9205; Cell Signaling Technology), total S6K (1 : 1000 dilution; Cat. No. sc‐230; Santa Cruz Biotechnology), phospho‐S6RP (1 : 1000 dilution, p‐Ser^240^ and p‐Ser^244^; Cat. No. 2215; Cell Signaling Technology), total S6RP (1 : 1000 dilution; Cat. No. sc‐74459; Santa Cruz Biotechnology), phospho‐4E‐BP1 (1 : 1000 dilution; p‐Ser^65^, Cat. No. 1365; Cell Signaling Technology), and tubulin α (1 : 2000 dilution; Cat. No. CP06‐100UG; Calbiochem, San Diego, CA, USA).

### 2.14 *In silico* coexpression analyses

Coexpression matrices were calculated from the corresponding expression matrices for the indicated genes using the *corrplot* package and the GEO dataset GSE78958 [40]. The statistical significance of each Pearson correlation coefficient (*r*) is indicated by the dot size and color.

### 2.15 Determination of MTT metabolization in cultured cells

We used the 3‐(4,5‐dimethylthiazol‐2‐yl)‐2,5‐diphenyl‐2H‐tetrazolium bromide (MTT) assay (Cat. No. M5655; Sigma‐Aldrich) to indirectly determine the metabolic growth properties of cell lines under study. To this end, cells were plated in 24‐well dishes (7000 cells per well) and cultured in RPMI supplemented with 10% fetal calf serum for the indicated periods. At the indicated time points, the culture medium of each well was discarded and replaced with 250 mL of MTT solution (0.5 mg·mL^−1^) in phosphate‐buffered saline solution. After 1 h at 37 °C in a 5% CO_2_ atmosphere, 500 mL of dimethyl sulfoxide (Cat. No. D8418; Sigma‐Aldrich) was added to each well to dissolve the formazan crystals formed. After 15 min, the absorbance at 570 nm wavelength was measured with a microplate reader Ultraevolution (Cat. No. 16129929; Tecan, Mannedorf, Switzerland).

### 2.16 Basal apoptotic rates

Assessment of the apoptotic index was made by flow cytometry using the Annexin V–VF blue apoptosis detection kit (Cat. No. ANXVKCFB7; ImmunoStep, Salamanca, Spain) following manufacturer's instructions. Data acquisition and analysis were done in a FACSAria III flow cytometer (BD Biosciences) and data analyzed using the flowjo software (10.8.2; FlowJo, Ashland, OR, USA). For each analysis, 50 000 events were acquired.

### 2.17 Ferroptosis assays

40 000 cells per P24 well of indicated 4T1 lines were seeded and grown overnight and, subsequently, treated with the indicated concentrations of ML162 (Cat. No. S4452; Selleckchem). After 24 h, cells were fixed with 10% formaldehyde for 10 min and stained for 10 min with Giemsa (Cat. No. GS80; Sigma‐Aldrich) diluted 1 : 7 in distilled deionized water. The solution was then removed, plates washed three times with distilled deionized water and air dried at room temperature. Stained cells were scanned using an EPSON V700 scanner (EPSON, Suwa, Nagano, Japan).

### 2.18 Determination of metabolic parameters

Oxygen consumption was measured in a Seahorse XFe24 Analyzer (Agilent, Santa Clara, CA, USA). To this end, 20 000 cells/well of the indicated 4T1 lines were grown overnight in fibronectin (2 μg·mL^−1^) pre‐coated Seahorse XF24 V7 PS Cell Culture Microplates (Cat. No. 102342‐100; Agilent). Prior to the assay, cells were washed and incubated for 1 h at 37 °C in a non‐CO_2_ incubator with Seahorse phenol red‐free RPMI (Cat. No. 103576‐100; Agilent) supplemented with 1 mm pyruvate (Cat. No. 103578‐100; Agilent), 2 mm glutamine (Cat. No. 103579‐100; Agilent), and 10 mm glucose (Cat. No. 103577‐100; Agilent). For the Mito Stress test, 1.5 μm oligomycin (Cat. No. 495455; Merck), 1 μm carbonyl cyanide‐p‐trifluoromethoxy phenyl‐hydrazone (FCCP; Cat. No. C2920; Sigma‐Aldrich), and a mix of 1 μm antimycin A (Cat. No. A8674‐50MG; Sigma‐Aldrich) plus 1 μm rotenone (Cat. No. R8875‐1G; Sigma‐Aldrich) were sequentially added to the cells. Compounds were diluted in the assay media and loaded into an overnight‐hydrated XFe24 sensor cartridge (Cat. No. 102342‐100; Agilent) using the Seahorse CF calibrant solution (Cat. No. 102342‐100; Agilent).

### 2.19 Animal‐based studies

*In vivo* work was performed exactly as described in a previous work from our laboratory [27]. In the case of breast cancer tumorigenesis analyses, 5 × 10^3^ cells were resuspended in 100 mL of phosphate‐buffered saline solution and injected into the fourth right mammary gland of female BALB/c mice (6–8‐week‐old). Tumor growth was analyzed by measuring tumor length, width, and depth to calculate the overall tumor volume. Metastatic dissemination was quantified both *de visu* and histologically (three sections per tumor) after sacrificing the mice 32 days after the orthotopic implantation.

In the case of long‐term lung colonization assays, cells were resuspended in phosphate‐buffered saline solution as described in the previous paragraph and injected into the tail vein (unless otherwise stated, 5 × 10^4^ cells were injected in each experiment). After 2 weeks, mice were euthanized, their chest cavity exposed through a midline chest incision, the trachea cannulated with a 20‐gauge caterer, and the lungs slowly inflated with 1 mL of India ink (1 : 16 dilution in phosphate‐buffered saline solution; Parker, Ville‐la‐Grande, France). Lungs were extracted and distained by immersion in Fekete's solution (100 mL of 70% ethanol [Cat. No. E7148; Sigma‐Aldrich], 10 mL of 4% formaldehyde [Cat. No. 3800750; Leica, Wetzlard, Germany], and 5 mL of 100% glacial acetic acid [Cat. No. 131008.1611; PanReac, Castellar del Valles, Barcelona, Spain]) and, subsequently, the metastatic nodules were visually counted. Alternatively, the lungs were extracted, fixed in 4% formaldehyde (Cat. No. 3800750; Leica), and embedded in paraffin for hematoxylin and eosin staining. In studies conducted with monocrotaline‐treated mice, animals were injected twice intravenously with 1.5 mg·mL^−1^ monocrotaline (Cat. No. PHL89251; Sigma‐Aldrich) the week before cancer cells were introduced as above in the tail vein. After 2 weeks, the animals were sacrificed and the lungs extracted as above.

To measure the extravasation of breast cancer cells, the indicated cell lines were labeled with 5 μm of a cell tracker (CellTracker Green carboxymethyl fluorescein diacetate; Cat. No. C2925; Invitrogen) for 1 h and then intravenously injected into mice (1 × 10^6^ cells per mouse). After 48 h, the mice were injected intravenously with a rhodamine‐conjugated lectin (Cat. No. RL‐1002‐25; Vector Laboratories, Newark, CA, USA) to stain lung capillaries and animals were sacrificed 1 h later. Lungs were then extracted, fixed in paraformaldehyde (Cat. No. 252931; PanReac), and examined by confocal fluorescence microscopy to determine the number of cancer cells (those exhibiting green fluorescence) present in the lung parenchyma (six confocal sections per tumor).

### 2.20 Immunostaining techniques

The immunohistochemical procedures presented in this work were carried out by the personnel of the Molecular Pathology Unit of our institution. Tissues were extracted, fixed in 4% paraformaldehyde, cut into 2–3‐mm‐thick sections, and stained with hematoxylin and eosin. For immunohistochemical staining, paraffin‐embedded sections were incubated with rabbit polyclonal antibodies to either Ki67 (1 : 100 dilution; Cat. No. KI67‐MM1‐L; Leica) or CD31 (1 : 50 dilution; Cat. No. 124432; Abcam). After an overnight incubation, the tissue slides were rinsed with phosphate‐buffered saline solution, incubated for 1 h at room temperature in a milk/phosphate‐buffered saline solution containing a goat anti‐rabbit immunoglobulin G–horseradish peroxidase conjugate (Cat. No. RPN4301; Cytiva), rinsed with phosphate‐buffered saline solution, and developed with diaminobenzidine (Cat. No. K3467; Dako, Glostrup, Denmark). The signals were quantified blindly with imagej software (version 1.53a, National Institutes of Health, Bethesda, MD, USA) using three sections per tumor. Apoptotic cells were detected using the TUNEL‐based *In Situ* Cell Detection Kit (Cat. No. 11684795910; Roche). To this end, sections were deparaffinized, hydrated, and digested with proteinase K (Cat. No. S3020; Dako) for 30 min at 37 °C and then subjected to the TUNEL staining according to the manufacturer's instructions. TUNEL‐positive cells were visually scored using a standard immunofluorescence microscope (CTR600; Leica).

### 2.21 Transcriptome profiling and bioinformatics

RNA was isolated using an RNeasy Mini Kit (Cat. No. 74104; Qiagen, Hilden, Germany) and analyzed using the Affymetrix platform (Clariom™ S Assay HT; Cat. No. 902971; ThermoFisher Scientific) at the CIC Genomics Core Facility according to the manufacturer's recommendations. r (version 3.6.3, R Foundation, Indianapolis, IN, USA) was used to perform the bioinformatic analyses. Signal intensity values were obtained from CEL files after applying the robust multichip average function from the Affy package for background adjustment, quantile normalization, and summarization [41]. Differentially expressed genes were identified using linear models for microarray data (limma) [42], and *P*‐values were adjusted for multiple comparisons by applying the Benjamini–Hochberg correction method (FDR) [43]. An FDR *q*‐value of 0.05 was set as the threshold for statistical significance. The heatmap3 package was used to generate the expression heatmaps for the indicated genes as indicated above. Gene Ontology (GO) and Kyoto Encyclopedia of Genes and Genomes (KEGG) pathway enrichment analyses were performed using DAVID (https://david.ncifcrf.gov). GSEA were performed with the described gene sets using gene set permutations (*n* = 1000) for the assessment of significance and signal‐to‐noise metrics for ranking genes [38]. Raw data from these microarray analyses were deposited in the GEO platform (GSE253308). The gene sets used in the GSEA analyses were obtained from both the GEO dataset GSE33348 [27] and the Molecular Signatures Database (MSigDB, v7.4) [44].

### 2.22 Bioinformatics analyses of tumor expression datasets

Survival analyses were performed using Kaplan–Meier estimates of distant recurrence‐free survival according to the level of enrichment of the described transcriptional signatures [45]. This enrichment was calculated using ssGSEA [38, 46], and Mantel–Cox test was subsequently applied to statistically validate the differences between the survival distributions [47]. The GEO accession codes for the human datasets used in this study were GSE25066 (for distant recurrence‐free survival analyses) [48, 49] and GSE2603 (for lung metastasis‐free survival analyses) [50].

### 2.23 Statistics

Student's *t*‐test (two groups) or one‐way ANOVA (more than two groups) were used to perform statistical analyses. The sample size and the number of independent experiments for each experiment are indicated in the appropriate figure legend. The experimental values in the graphs are provided as the means and SEM. Results with *P*‐values ≤ 0.05 were considered to indicate statistical significance.

## 3 Results

### 3.1 VAV‐regulated expression of mevalonate pathway transcripts in 4T1 cells

**Fig. 1:**
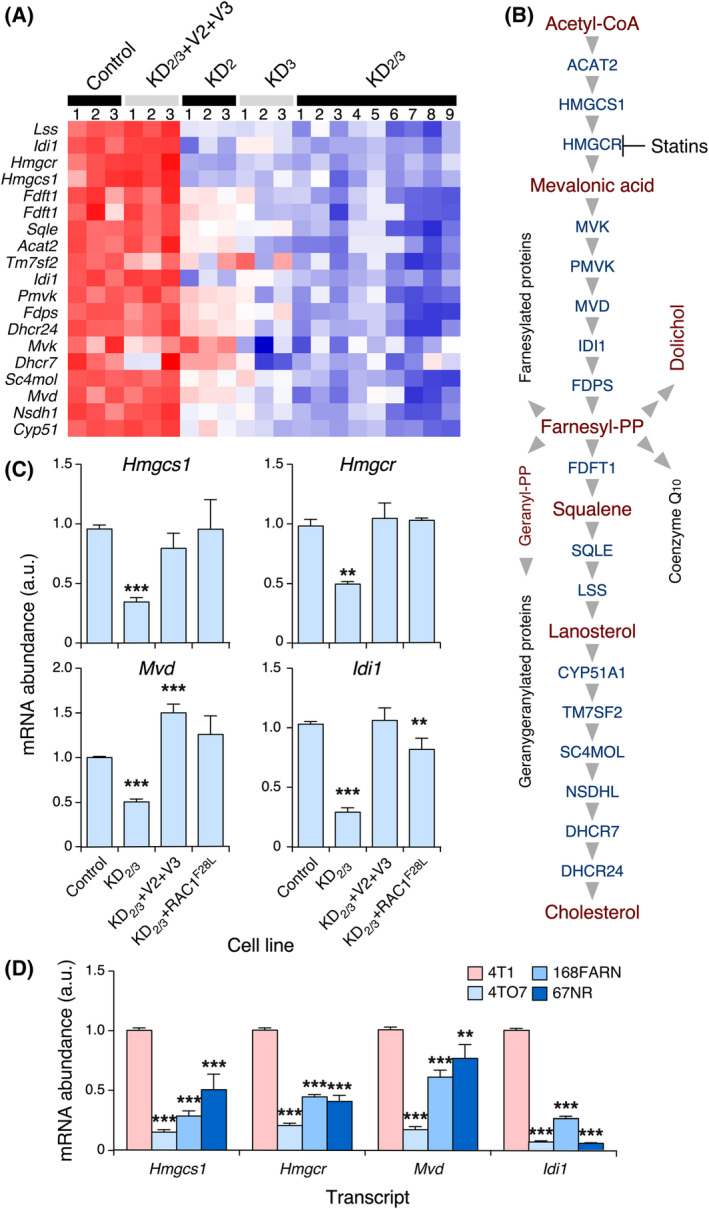
VAV‐regulated expression of mevalonate pathway genes in 4T1 cells. (A) Heatmap showing the expression of mevalonate pathway genes (left) in the indicated cell lines (top). Genes upregulated and downregulated are shown in red and blue colors, respectively. The gradient reflects the relative fold‐change variation in the expression of the genes of interest. The replicates for each cell line are shown at the top. Control, 4T1 cells transduced with the empty pLKO vector. (B) Schematic representation of the mevalonate pathway. Enzymes whose mRNA expression is downregulated in *Vav* knockdown 4T1 cells are shown in blue. The main metabolites of the pathway are shown in red. (C) Abundance of the interrogated transcripts (top) in the indicated 4T1 cell derivatives (bottom). The values are shown relative to the abundance of each transcript in the control cell line, which was given an arbitrary number of 1. a.u., arbitrary units. (D) Abundance of the interrogated transcripts (bottom) in the indicated breast cancer cell lines (inset). The values are shown relative to the abundance of each transcript in the control cell line, which was given an arbitrary number of 1. Data shown in panels C and D represent the means ± SEM. **P ≤ 0.01; ***P ≤ 0.001 relative to the control cell line (Student's *t*‐test, *n* = 3 independent experiments in each case).

In previous publications [27, 28], we demonstrated that the endogenous VAV2 and VAV3 proteins control a wide distal transcriptional program that is important for both the *in vivo* tumorigenic and metastatic properties of this cell line. This led us to identify several VAV2;VAV3‐regulated distal elements that are involved in specific stages of the metastatic program of breast cancer cells to the lung [27, 28]. Further functional annotation of the *Vav2*;*Vav3*‐dependent 4T1 cell transcriptome revealed that the elimination of endogenous *Vav2* or *Vav3* mRNAs and, to a greater extent, the combined depletion of both transcripts led to a marked reduction in the expression of all transcripts encoding the main enzymes involved in the mevalonate pathway and its main anabolic branches (Fig. 1A,B). Normal expression of all these transcripts was restored upon the reexpression of the wild‐type versions of VAV2 and VAV3 in double *Vav2;Vav3* knockdown 4T1 cells (from now on, designated as KD_2/3_ cells) (Fig. 1A), demonstrating that this metabolic pathway is directly associated with the proper activity of these GEFs in 4T1 cells. Using quantitative reverse transcription polymerase chain reaction (qRT–PCR) analyses, we confirmed a reduction in the abundance of several of the relevant transcripts of this metabolic pathway (*Hmgcs1*, *Hmgcr*, *Mvd*, *Idi1*) in KD_2/3_ cells (Fig. 1C). Furthermore, and as previously shown with the microarray data (Fig. 1A), we found that the levels of these transcripts were fully restored upon reexpression of VAV2 and VAV3 in KD_2/3_ cells (Fig. 1C). This effect is probably catalysis‐dependent because the expression of the transcripts of these mevalonate pathway enzymes could also be restored upon the stable expression of a gain‐of‐function mutant (F28L) of the GTPase RAC1 in KD_2/3_ cells (Fig. 1C).

These observations suggested that changes in the functional status of the mevalonate pathway could contribute to the defective tumorigenic and metastatic features previously found in 4T1 KD_2/3_ cells. Consistent with this hypothesis, we observed that the expression of many mevalonate pathway‐related transcripts was higher in metastatic (4T1 and 66c14) breast cancer cell lines than in nonmetastatic ones (4TO7 168FARN and 67NR) (Fig. S1 and Fig. 1D). In particular, the 4TO7 cell line exhibited the most consistent downregulation of *Hmgcs1*, *Hmgcr*, and *Idi1* mRNAs according to our qRT–PCR experiments (Fig. 1D). Notably, each of these nonmetastatic cell lines has defects in specific metastatic steps: migration from the primary tumor (67NR), metastatic spread further than the lymph nodes (168FARN), and survival upon reaching the lung parenchyma (4TO7 cells) [29].

### 3.2 VAV‐mediated expression of mevalonate pathway genes is RAC1‐ and SREBF‐dependent

**Fig. 2:**
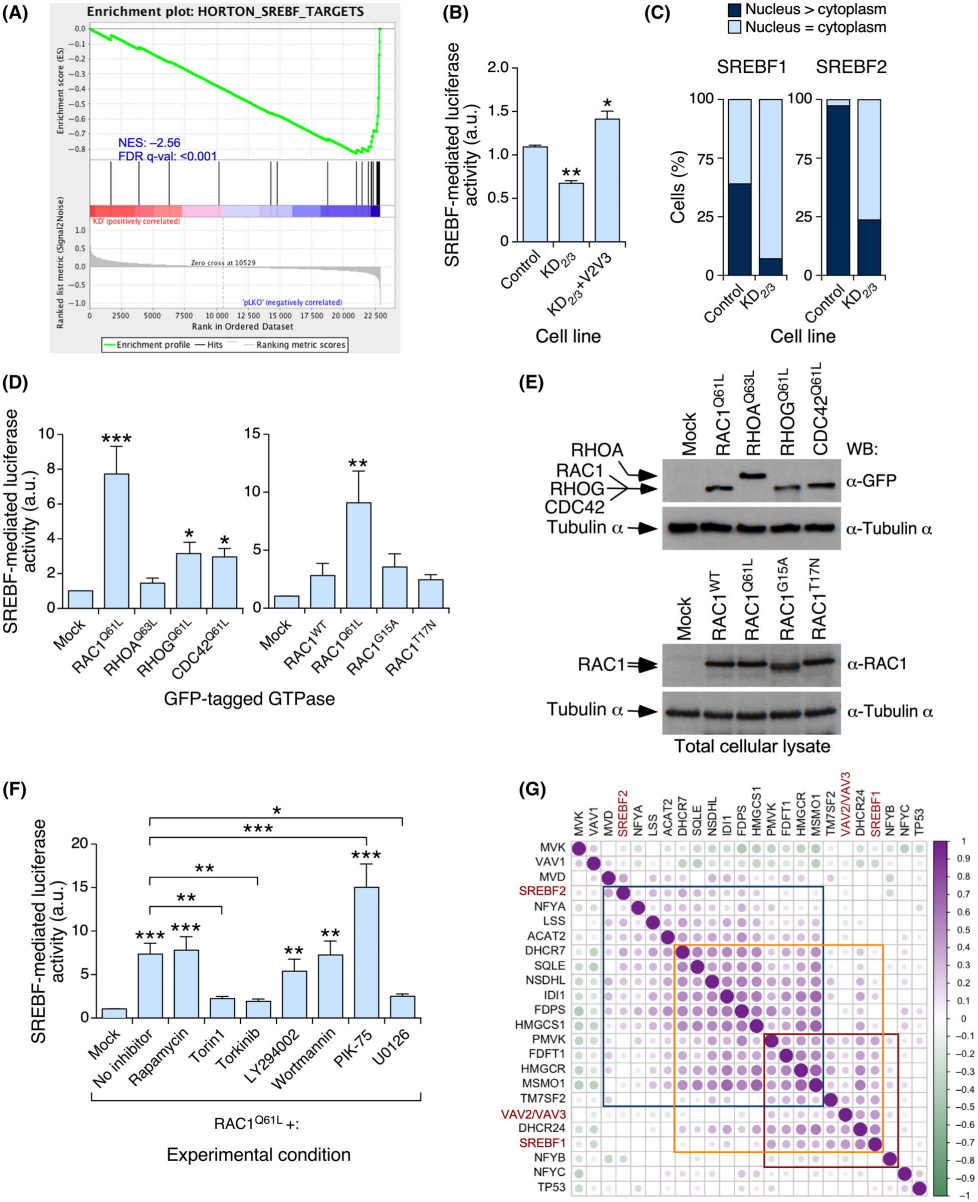
VAV‐mediated expression of mevalonate pathway genes is RAC1‐ and SREBF‐dependent. (A) GSEA showing the loss of the SREBF gene signature in *Vav2*;*Vav3* knockdown 4T1 cells. The normalized enrichment score (NES) and false discovery rate values (FDR *q*‐value) are indicated inside the GSEA graph. (B) SREBF activity in the indicated 4T1 cell lines determined using a luciferase reporter assay. Values are shown relative to the value obtained in the control cell line, which was given an arbitrary value of 1. (C) Quantitation of the subcellular distribution of SREBFs in indicated cell derivatives (bottom) using confocal microscopy analyses (*n* = 3 independent experiments). (D) SREBF activity in parental 4T1 cells upon the transient expression of the indicated GTPases (bottom). In the left panel, transfections were made with expression vectors encoding the indicated EGFP‐tagged GTPases. In the right panel, transfections were made with untagged RAC1 versions. The values are shown relative to the values obtained in the mock‐transfected samples, which were given an arbitrary value of 1. (E) Western blot showing the expression of the indicated GTPases (first and third panels from top) in the experiment shown in D. Equal loading was demonstrated using immunoblotting with antibodies to tubulin α (second and fourth panels from top). (F) Luciferase reporter experiments showing the effect of indicated inhibitors (bottom) in the RAC1^Q61L^‐mediated activation of SREBF. (G) Expression correlation matrix of the indicated transcripts using the human breast tumor samples from the GEO microarray dataset GSE65194. Positive and negative correlations are shown in purple and green, respectively. The size and color intensity are proportional to the Pearson correlation coefficient found for each transcript pair. Boxes indicate groups of genes whose expression is more tightly coregulated. Data shown in panels B, D, and F show the means ± SEM. *P ≤ 0.05; **P ≤ 0.01; ***P ≤ 0.001 relative to the control using either the Student's *t*‐test (B, D) or the one‐way ANOVA test (F) (*n* = 3 independent experiments in each case).

We next investigated the VAV‐dependent mechanism involved in the regulation of the mevalonate pathway in breast cancer cells. Based on our results shown in Fig. 1C, we surmised that this response was dependent on the VAV catalysis‐mediated stimulation of the RAC1 GTPase because normal expression of mRNAs for mevalonate pathway components is rescued upon expression of a fast‐cycling mutant of RAC1 (F28L) in KD_2/3_ cells. We also assumed that this pathway must involve the stimulation of SREBFs, a family of transcription factors of the basic‐helix–loop–helix leucine zipper subtype that regulates the expression of mevalonate pathway genes [4, 13, 51], because we found using *in silico* gene set enrichment analyses (GSEA) that the SREBF gene signature was downregulated in KD_2/3_ 4T1 cells compared with controls (Fig. 2A). Consistent with this idea, we observed using luciferase reporter assays with a plasmid bearing an SREBF responsive promoter that the endogenous activity of this transcription factor was reduced in KD_2/3_ cells (Fig. 2B). This defect was eliminated upon the expression of both VAV2 and VAV3 in these knockdown cells (Fig. 2B), further indicating the VAV dependency of this readout in 4T1 cells. In line with this, we observed using confocal microscopy that the endogenous SREBF proteins were less concentrated in the nuclei of KD_2/3_ cells than in the case of control cells (Fig. 2C). Using the luciferase reporter assay described above, we also found that an active version of RAC1 (Q61L mutant) triggered high levels of activity in endogenous SREBFs when transiently expressed in parental 4T1 cells (Fig. 2D,E). This response was not observed or was severely reduced when cells were transiently transfected with gain‐of‐function mutants of other RHO family GTPases such as RHOA^Q63L^, RHOG^Q61L^, or CDC42^Q61L^ (Fig. 2D,E). Likewise, it was not induced when cells were transfected with wild‐type or dominant negative mutants of RAC1 (Fig. 2D,E). Taken together, these findings indicate that the VAV–RAC1–SREBF axis is likely involved in the regulation of mevalonate pathway genes in 4T1 breast cancer cells.

Further experiments indicated that the RAC1^Q61L^‐mediated activation of SREBF‐mediated luciferase activity was not affected by the addition of some specific mTORC1 (rapamycin) or PI3K inhibitors to the transfected 4T1 cells (Fig. 2F). In fact, one of the PI3K inhibitors (PIK‐75) led to a more robust SREBF‐mediated luciferase activity when compared to the untreated, RAC1^Q61L^‐expressing cells (Fig. 2F). In contrast, dual inhibitors for mTORC1 and mTORC2 (torin1, torkinib) totally abrogated the stimulation of SREBF transcriptional activity by RAC1^Q61L^ in the same experimental setting (Fig. 2F). These inhibitors, but not rapamycin, also reduced the mRNA levels for HMGCR (Fig. S2A) and SREBP2 (Fig. S2B) in control 4T1 cells. We also observed that the inhibition of MEK induced a similar reduction in RAC1^Q61L^‐induced SREBF transcriptional activity (Fig. 2F). Involvement of MEK–ERK signaling in the regulation of SREBFs has been demonstrated before in hepatocarcinoma cells [52, 53]. Western blot analyses confirmed that the inhibitors for PI3K used in these experiments effectively abrogated the activation of AKT (Fig. S2C). Likewise, the MEK inhibitor promoted a reduction and upregulation in the phosphorylation of ERK and AKT, respectively (Fig. S2C). We also observed that the torin1 and torkinib inhibitors inhibited downstream PI3K signaling elements at the specific concentrations that led to the abrogation of RAC1^Q61L^‐induced SREBF transcriptional activity and the reduction in *Hmgcr* and *Srebp2* mRNA levels (Fig. S2D). More importantly, we found that torin1 and torkinib, but not rapamycin, could effectively block the phosphorylation of 4E‐BP1 (Fig. S2D, ninth panel from top), as previously described in other experimental settings [4, 54]. These results indicate that the effect of RAC1^Q61L^ on SREBP activity is probably mediated by rapamycin‐insensitive mTORC1/2 downstream elements and MEK–ERK signaling. In contrast, it seems to be independent of PI3K and rapamycin‐sensitive mTORC1 pathways.

Using *in silico* coexpression matrix analyses with publicly available gene expression datasets from human tumors, we found that the combined expression levels of *VAV2* and *VAV3* transcripts also positively correlated with the abundance of a subset of transcripts for mevalonate pathway enzymes (*HMGCR*, *PMVK*, *FDFT1*, *TM7SF2*, *SC4MOL*, *DHCR24*) and the *SREBF1* mRNA in human breast cancer samples (Fig. 2G, genes included in the red box). In contrast, there was less overlap with the subset of mevalonate pathway genes whose expression was directly correlated with *SREBF2* mRNA levels (Fig. 2G, genes included in the blue box). No significant correlation of any of these two mevalonate pathway‐related gene subsets was found with the mRNA for *TP53*, a suppressor gene that has been associated with the regulation of the mevalonate pathway in cancer cells [4, 5, 55, 56, 57].

### 3.3 HMGCS1 and HMGCR are important for primary tumorigenesis

To investigate the potential role of this metabolic pathway in the tumorigenic phenotype of 4T1 cells, we next generated two independent knockdown 4T1 cell derivatives for HMGCS1 (KD_*Hmgcs1*_–1.9 and KD_*Hmgcs1*_–2.7) and HMCGR (KD_*Hmgcr*_–3.2 and KD_*Hmgcr*_–5.4) (Fig. S3A). These two knockdown transcripts encode enzymes that are located in the early steps of this metabolic pathway (see above, Fig. 1B). In addition, the HMGCR enzyme is directly responsible for the production of mevalonate from 3‐hydroxy‐3‐methylglutaryl‐coenzime A as well as the molecular target for statins [3, 4]. Interestingly, the depletion of the *Hmgcs1* transcript promoted the upregulation of other mRNAs encoding mevalonate pathway components such as *Hmgcr*, *Mvd*, and *Idi1* (Fig. S3B). This result suggests the potential existence of compensatory mechanisms that counteract the reduction in HMGCS1 activity in 4T1 cells. This compensatory mechanism was much less apparent in *Hmgcr* knockdown cells, as we only detected statistically significant changes in the expression of the *Mvd* transcript (Fig. S3C).

Unlike the case of the *Vav2;Vav3* knockdown cells [27, 28], we observed that the downregulation of either of those two mRNAs did not change the morphology of 4T1 cells compared with that of controls (Fig. S3D). However, the downregulation of the *Hmgcr* mRNA, but not of the *Hmgcs1* transcript, led to a small but statistically significant decrease in the metabolization of MTT in 4T1 cells in 2D cultures that was similar to what was previously found in KD_2/3_ cells [27] (Fig. S3E). The effect of the depletion of these two enzymes was also very week in basal apoptosis (Fig. S3F). In contrast, we found that the *Hmgcr* knockdown cells, but not the *Hmgcs1* knockdown cells, were more sensitive to the induction of ferroptosis upon treatment with a well‐known inhibitor of glutathione peroxidase 4 (ML162) (Fig. S3G). Finally, we observed using Seahorse‐based metabolic experiments (Fig. S4A,B) that *Hmgcs1* knockdown cells, and to a larger extent the *Hmgcr* knockdown cells, were deficient in key mitochondrial metabolic parameters such as basal respiratory, maximal respiration, spare respiratory capacity, ATP production, and proton leak rates (Fig. S4C,D).

**Fig. 3:**
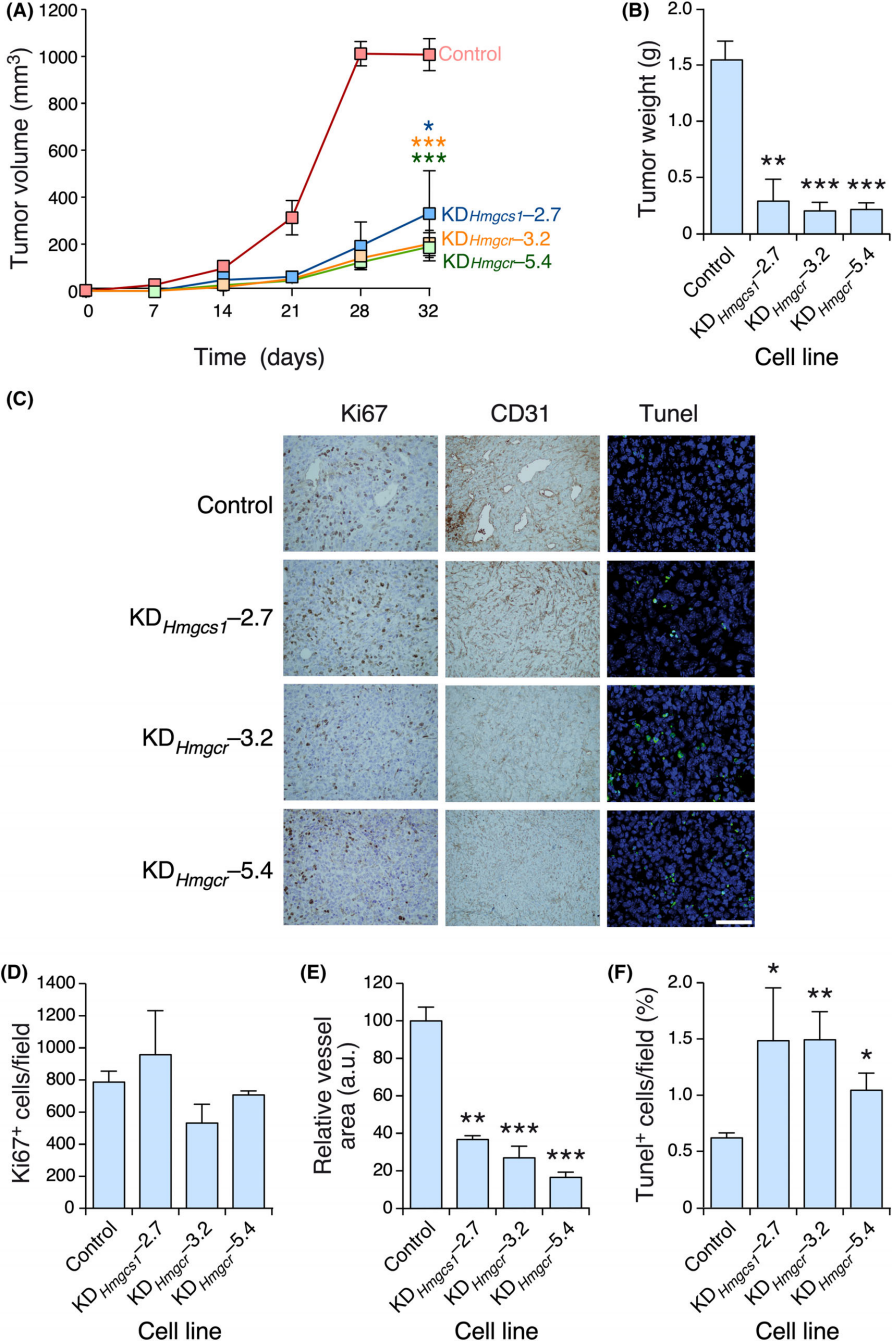
HMGCS1 and HMGCR are important for primary tumorigenesis. (A, B) Growth kinetics (A) and final weight (B) of tumors generated by the indicated cell lines. Statistics were performed at the 32‐day time point. (C) Representative images of immunostained sections used to evaluate the proliferation (Ki67^+^ cells), angiogenesis (CD31^+^ cells), and apoptosis (Tunel^+^ cells) parameters of the tumors in panel (A). Scale bar, 50 μm. (D–F) Quantification of the proliferation (Ki67^+^ cells, D), angiogenesis (CD31^+^ cells, E), and apoptosis (Tunel^+^, F) in the experiments performed in C. Data shown in panels A, B, and D–F represent the means ± SEM. *P ≤ 0.05; **P ≤ 0.01; ***P ≤ 0.001 relative to control tumors using the Student's *t*‐test. In (A, B), *n* = 5 animals for each cell type, 1 independent experiment. In (D–F), *n* = 3 lung sections per mice, 5 animals in each experimental condition, 1 independent experiment.

To investigate the effect of these knockdowns *in vivo*, we next orthotopically implanted *Hmgcs1*‐ or *Hmgcr*‐depleted cells in the mammary fat pads of recipient female mice. In contrast to the minor effects found on proliferation in the cell culture experiments, we observed that *Hmgcs1* or *Hmgcr* knockdown 4T1 cells could form primary tumors with lower efficiency than controls (Fig. 3A,B). Histological studies revealed that the deficiencies in primary tumor development in these knockdown cell lines were not caused by *in vivo* proliferation defects, as inferred from the lack of statistically significant variations in Ki67 immunostaining in the tumors formed by the knockdown and control cells (Fig. 3C,D). In contrast, *Hmgcs1* and *Hmgcr* knockdown cells generated tumor masses that were less angiogenic (Fig. 3C,E) and more prone to basal apoptosis (Fig. 3C,F) than controls.

### 3.4 HMGCR is important for lung metastasis

**Fig. 4:**
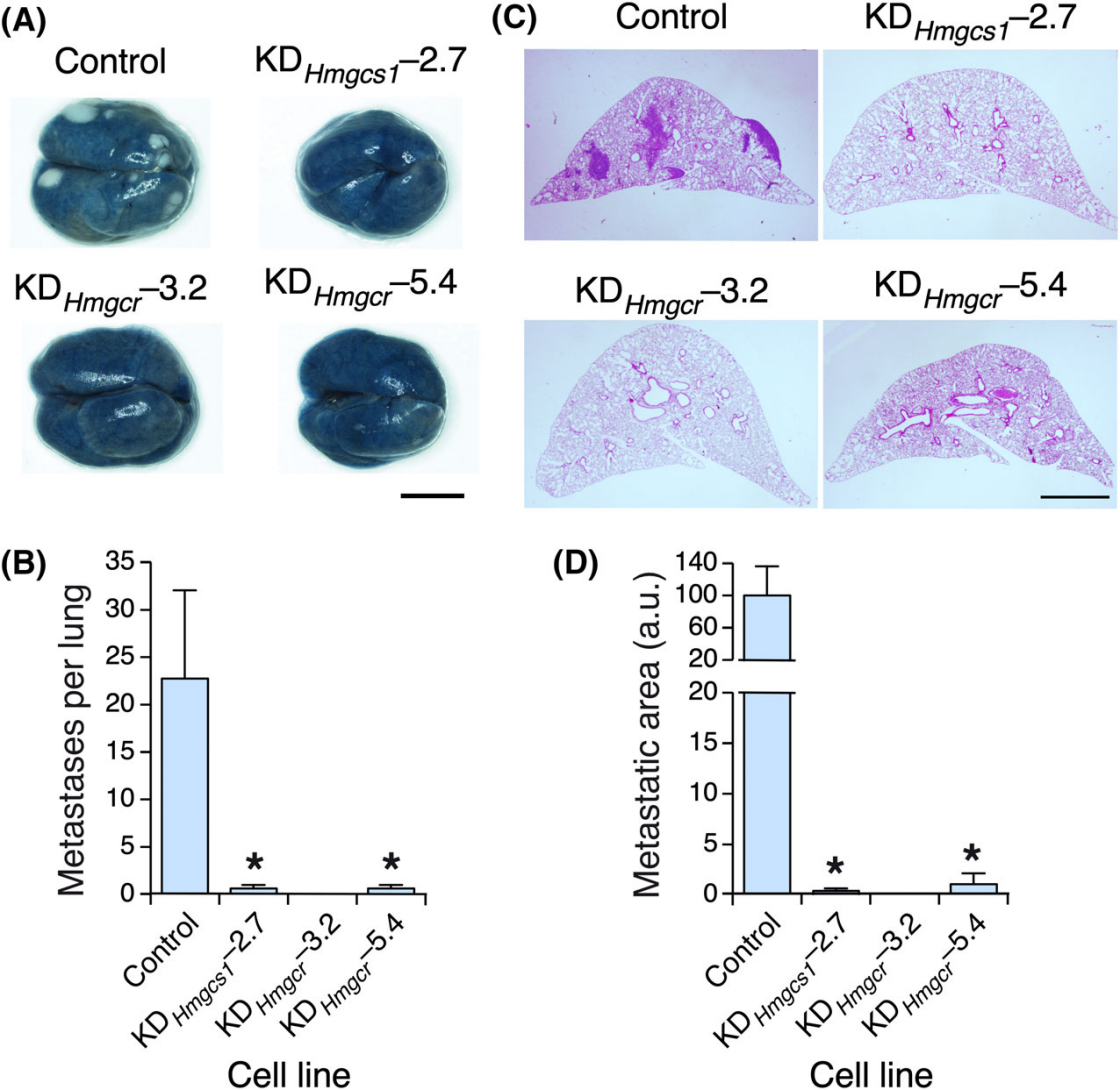
HMGCR is important for lung metastasis. (A, B) Representative image (A) and quantification (B) of the metastases found in tumor‐bearing animals orthotopically transplanted with the indicated cell lines. In A, scale bar = 500 μm. (C, D) Representative image (C) and quantification (D) of the size of metastases found in the experiments shown in A and B. In (C), scale bar = 1 mm. Data shown in panels B and D represent the means ± SEM. *P ≤ 0.05 relative to the control using the Student's *t*‐test. In (A), *n* = 5 animals per condition, 1 independent experiment. In (C), *n* = 3 lung sections per mice, 5 animals in each experimental condition, 1 independent experiment.

**Fig. 5:**
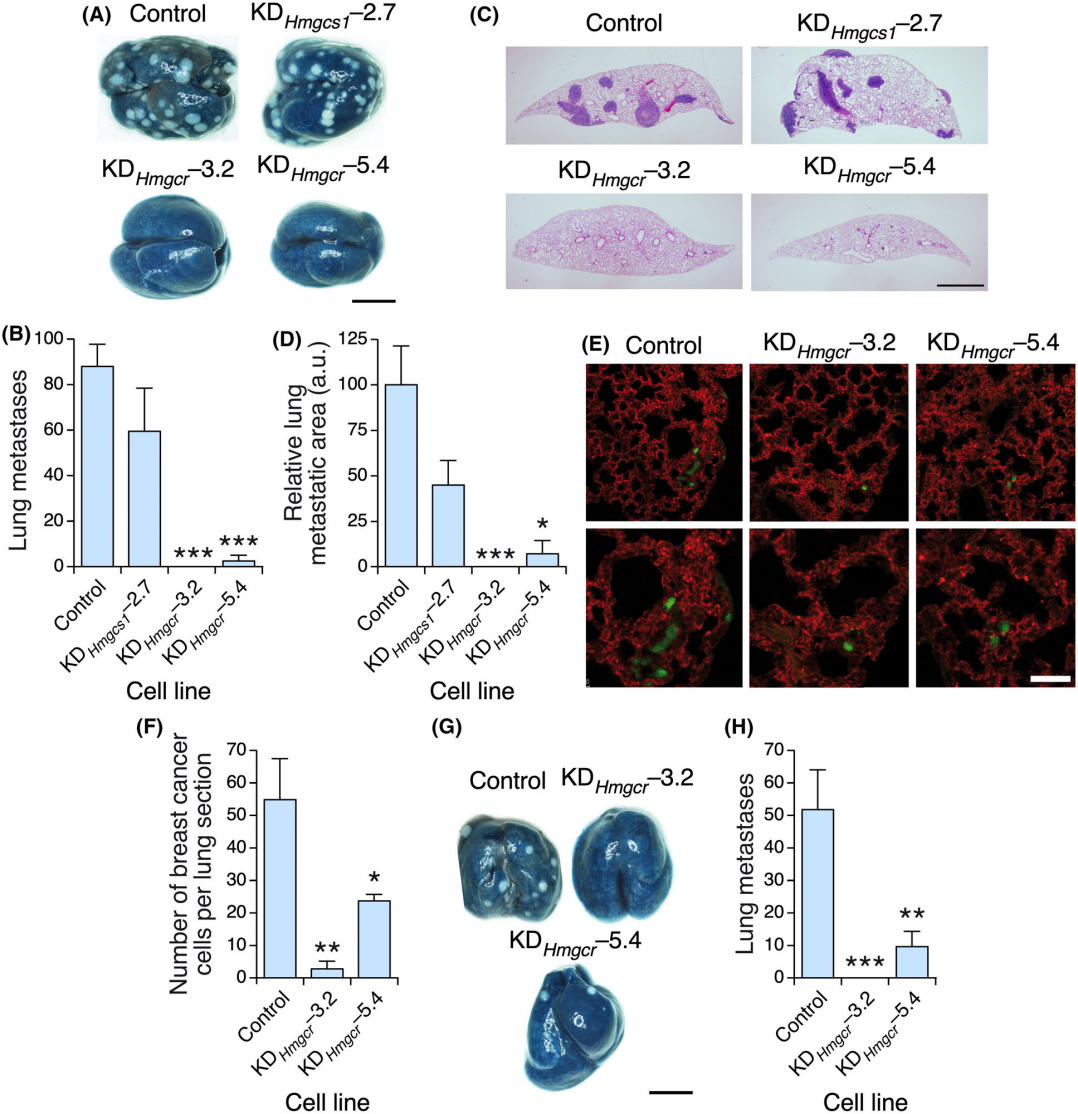
HMGCR is important for the extravasation of 4T1 cells and their fitness in the lung parenchyma. (A, B) Representative image (A) and quantification (B) of the metastases formed by the indicated intravenously injected 4T1 cells. In (A), scale bar = 500 μm. (C, D) Representative image (C) and quantification (D) of the size of the metastasis found in panel A. In (C), scale bar = 1 mm. (E, F) Representative image (E) and quantification (F) of the experiments carried out to measure the extravasation rates of the indicated cell lines into the lung parenchyma. In (E), scale bar = 25 μm. (G, H) Representative image (G) and quantification (H) of the metastases generated by the indicated cell lines upon being intravenous injected into monocrotaline‐treated mice. In (G), scale bar = 500 μm. Data shown in panels B, D, F, and H represent the means ± SEM. *P ≤ 0.05; **P ≤ 0.01; ***P ≤ 0.001 relative to the control using the Student's *t*‐test. In (A, B, G, H), *n* = 5 animals per experimental condition, 1 independent experiment. In (D, F), *n* = 3 lung sections per mice, 5 animals in each experimental condition, 1 independent experiment.

We also found in the proceeding *in vivo* experiments that the tumors from the *Hmgcs1* or *Hmgcr* knockdown cells generated fewer (Fig. 4A,B) and smaller (Fig. 4C,D) lung metastases than those from control 4T1 cells. To determine whether this effect was due to reduced primary tumorigenesis and/or to intrinsic defects in the metastatic properties of these cells, we intravenously injected control and knockdown cells to directly generate metastatic nodules in the lung. We found that the *Hmgcr*‐depleted cells exhibited severe metastatic defects compared with their control counterparts (Fig. 5A–D), indicating that they had impaired metastatic features. In contrast, we did not find statistically significant metastatic defects in *Hmgcs1* knockdown cells under the same experimental conditions (Fig. 5A–D). Thus, the reduced number of metastatic nodules formed by these cells in the orthotopic transplants is likely a mere reflection of their primary tumorigenesis defects (Fig. 3A,B).

These results suggest that the depletion of HMGCR affects the extravasation and/or implantation properties of 4T1 cells in the lung parenchyma. To investigate potential defects in the extravasation step, we intravenously injected control and knockdown cells that were preincubated with a cell‐permeable green chromophore to facilitate detection in the lung parenchyma 48 h after the extravasation step using confocal microscopy. Before euthanasia, we stained the lung capillaries with a rhodamine‐conjugated lectin to visualize cancer cells both in the capillaries and in the lung parenchyma. We found that the injected KD_*Hmgcr*_–3.2 and KD_*Hmgcr*_–5.4 cells, unlike the control cells, could not efficiently extravasate to the lung parenchyma (Fig. 5E,F). To evaluate whether these cells could also have survival defects upon extravasation, we next intravenously injected the interrogated cell lines into recipient mice that were pretreated with monocrotaline. This toxin increases the permeability of the lung vascular endothelium [58] and, therefore, facilitates colonization of the lung parenchyma by cells that are defective in the extravasation step [27, 59]. We observed that *Hmgcr* knockdown cells could not efficiently form metastatic nodules in the lungs of the recipient mice (Fig. 5G,H). Collectively, these results indicate that HMGCR is required for both the extravasation and survival of 4T1 cells in the lung niche.

### 3.5 HMGCR affects gene expression programs associated with different cancer hallmarks

**Fig. 6:**
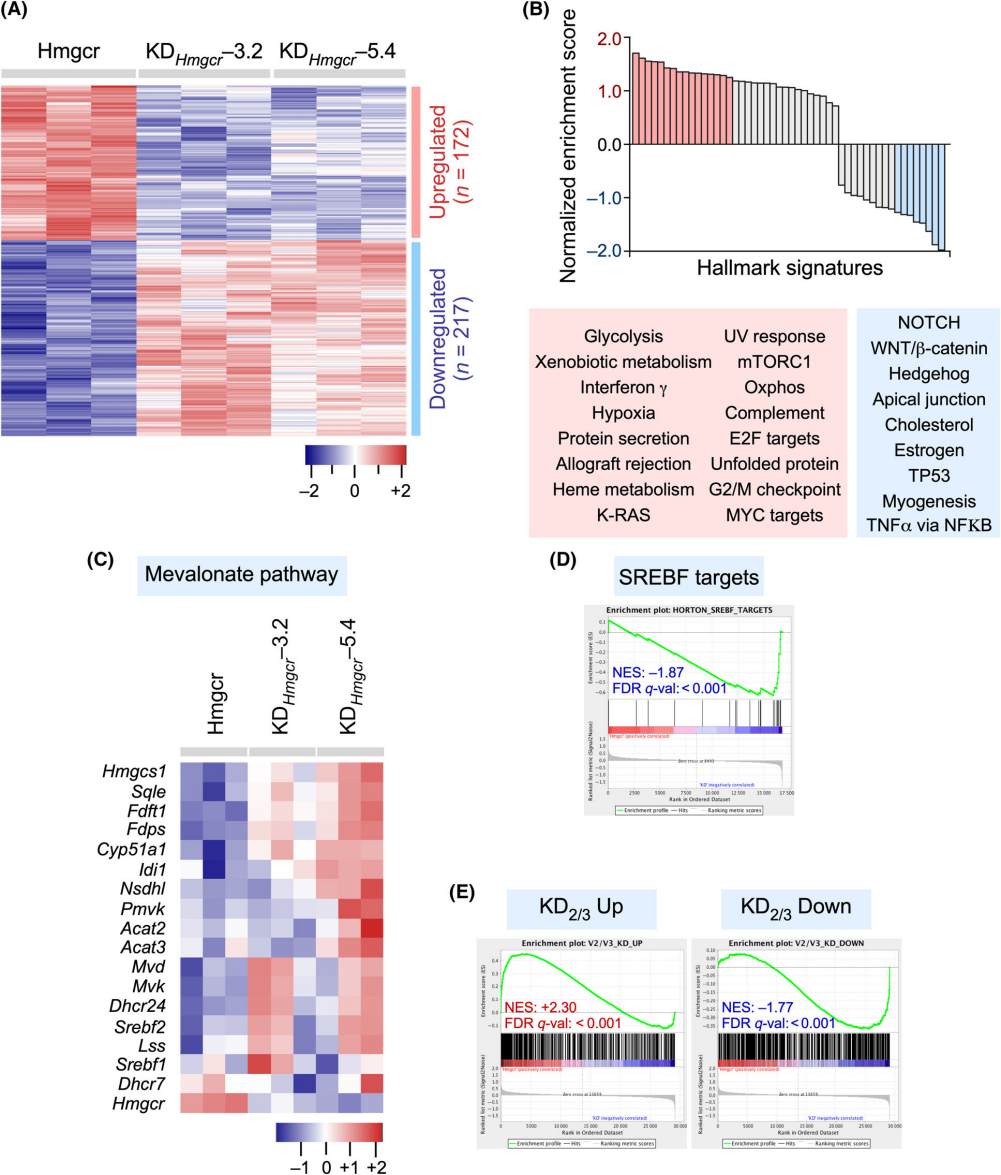
HMGCR affects gene expression programs associated with different cancer hallmarks. (A) Heatmap showing the transcripts whose expression was upregulated (red) or downregulated (blue) in the indicated 4T1 cell lines (top). Triplicates for each cell line (columns) are shown. Relative changes in abundance are shown in color gradients according to the scale shown at the bottom. (B) Main functional categories encoded by the upregulated (red) and downregulated (blue) HMGCR‐dependent transcripts. (C) Heatmap showing the expression of the genes of the mevalonate pathway in the indicated cell lines. Triplicates for each cell line (columns) are shown. The color codes are the same as those used in panel A. (D) GSEA showing the reduced expression levels of the SREBF gene signature in HMGCR‐overexpressing 4T1 cells. (E) GSEA showing the overlap between the VAV2;VAV3‐ and HMGCR‐dependent transcriptomes.

The protumorigenic and prometastatic effects of the mevalonate pathway described above led us to characterize the biological programs that are controlled by this metabolic route in breast cancer cells. To this end, we generated an HMGCR‐overexpressing 4T1 cell derivative and subsequently performed genome‐wide expression analyses to identify genes that, when compared with those in control cells, were regulated in opposite directions in HMGCR‐overexpressing versus *Hmgcr* knockdown 4T1 cells. We found 443 genes (thereafter referred to as the “HMGCR‐dependent gene signature”) that fulfilled these criteria (Fig. 6A). GSEA‐based annotation studies revealed that this gene signature is related to the expression of gene sets associated with proliferation‐related processes (K‐RAS, mTORC1, E2F, MYC, the G_2_/M checkpoint, ultraviolet response), basic metabolic programs (mTORC1, glycolysis, oxidative phosphorylation, xenobiotic responses, hypoxia, heme metabolism, protein secretion), and immune responses (interferon γ, allograft rejection, complement) (Fig. 6B and Fig. S5). The reduction of oxidative phosphorylation‐related gene sets agrees with our previous metabolic experiments performed in *Hmgcr* knockdown cells (Fig. S4). In addition, we found that depletion and overexpression of HMGCR correlated with reduced (Fig. S6A) and increased (Fig. S6B) levels of PI3K signaling in insulin growth factor 1‐stimulated 4T1 cells, respectively. We also found that the downregulated gene sets were linked to specific signaling responses (estrogens, Hedgehog, NOTCH, TP53, tumor necrosis factor α, WNT–β‐catenin) and biological programs (apical junctions, myogenesis) (Fig. 6B and Fig. S7). Interestingly, the overexpression and depletion of HMGCR promoted the downregulation and upregulation of some enzymes of the mevalonate pathway, respectively (Fig. 6B,C). This phenomenon was associated with the parallel downregulation of the SREBF target signature in HMGCR‐overexpressing cells (Fig. 6D). This is probably a consequence of the activation of compensatory mechanisms to maintain an adequate metabolic output from this route. Further GSEA analyses indicated a significant level of overlap between the HMGCR‐ and VAV2;VAV3‐dependent gene signatures in 4T1 cells (Fig. 6E), indicating a close functional association between these metabolic and signaling pathways.

### 3.6 The HMGCR‐dependent gene signature has prognostic value

**Fig. 7:**
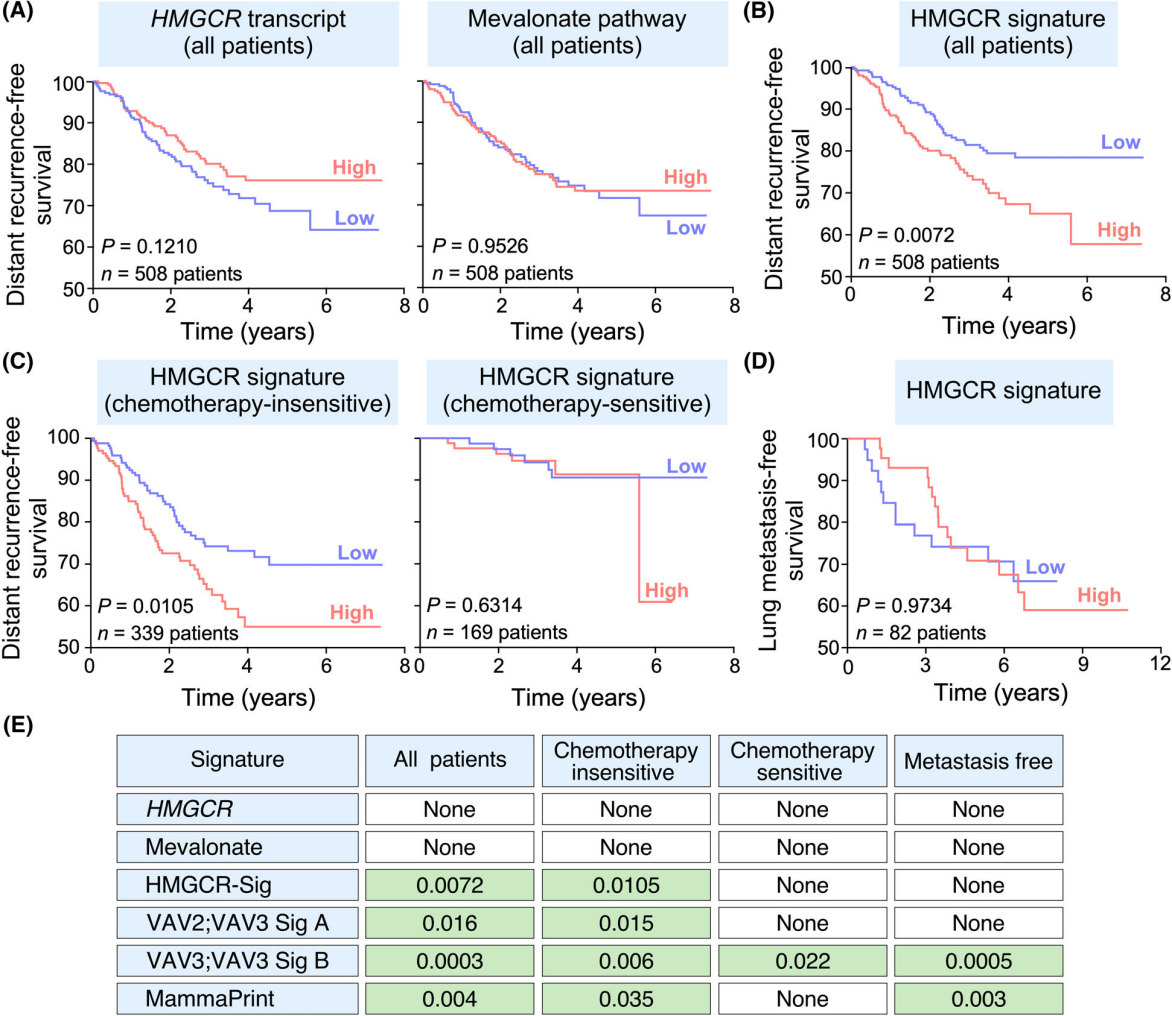
The HMGCR‐dependent gene signature has prognostic value. (A, B) Kaplan–Meier distance recurrent‐free survival according to the expression levels of the *HMGCR* transcript (A, left panel), mevalonate pathway enzyme‐encoding genes (A, right panel) or the HMGCR gene signature (B). High and low levels of expression are depicted in red and blue color, respectively. The Mantel–Cox *P*‐value and the number of patients are also indicated inside the graphs. The datasets used in these analyses were GSE25066 and GSE2603. (C, D) Kaplan–Meier distant recurrent‐free survival (C) and lung metastasis‐free survival (D) plots according to the enrichment of the HMGCR‐dependent gene signature in the indicated patient cohorts (top). The Mantel–Cox *P*‐value and the number of patients are indicated inside the graphs. The datasets used are those indicated above. The datasets used in these analyses were GSE25066 and GSE2603 (for survival analyses) and GSE2603 (for lung metastasis‐free survival analyses). (E) Prognostic value of the indicated gene signatures. The specific clinical parameters for which the interrogated gene signatures showed prognostic value are shaded in green. In this case, the Mantel–Cox *P*‐value is indicated.

We finally investigated the prognostic value of the *HMGCR* transcript levels and of gene signatures directly associated with either the mevalonate pathway or the HMGCR‐dependent gene transcriptome. As a control, we used three previously described gene signatures that exhibit different stratification features when used in breast cancer patients: (a) VAV2;VAV3‐dependent “Signature A” (109 gene probe sets), which is directly related to the acquisition of a mesenchymal state by 4T1 cells [28]; (b) VAV2;VAV3‐dependent “Signature B” (120 gene probe sets), which encompasses genes whose expression is directly correlated with the capacity of 4T1 cells to form lung metastases [27]; and (c) the MammaPrint gene signature, which is widely used in the clinic (70 genes) [60]. To this end, these signatures were used to interrogate microarray‐derived datasets from breast cancer patients that contained information about overall patient survival, chemoresistance‐related disease outcome, and/or lung metastasis‐free survival (see Section 2). We found that the abundance of either *HMGCR* mRNA (Fig. 7A, left panel, *P* = 0.121, *n* = 508 patients) or the entire set of mevalonate pathway‐related genes (Fig. 7A, right panel, *P* = 0.9526, *n* = 508 patients) did not have any stratification power in breast cancer patients. In contrast, the HMGCR‐related gene signature stratified breast cancer patients according to overall survival (Fig. 7B, *P* = 0.0072, *n* = 508 patients) or disease recurrence in chemotherapy‐insensitive patients (Fig. 7C, left panel; *P* = 0.0105, *n* = 339 patients). However, there was no predictive power in the case of disease recurrence when chemotherapy‐sensitive patients were interrogated (Fig. 7C, right panel; *P* = 0.6314, *n* = 169 patients) or for predicting metastasis development (Fig. 7D, *P* = 0.9734, *n* = 82 patients). This prognostic power was similar to that of the VAV2;VAV3‐dependent “Signature A” and the MammaPrint gene set in relation to overall survival (*P* = 0.0072 vs. 0.016 and 0.004, respectively), disease outcome in chemotherapy‐insensitive (*P* = 0.0105 vs. 0.015 and 0.035, respectively), and chemotherapy‐sensitive (no stratification power by any of them) of breast cancer patients (Fig. 7E). However, it was less efficient than MammaPrint in predicting lung metastasis‐free survival (no stratification power vs. *P* = 0.003) (Fig. 7E). The HMGRC‐dependent gene signature (as well as the VAV2;VAV3‐dependent “Signature A” and MammaPrint gene set) was much worse than the VAV2;VAV3‐dependent “Signature B,” as this latter gene set has stratification power for all the clinical parameters surveyed in these analyses (Fig. 7E). The best stratification power of this latter signature is probably due to its functional association with the tumorigenic and lung‐associated metastatic properties of breast cancer cells that have been previously described [27].

## 4 Discussion

In this study, we demonstrated that the VAV family members VAV2 and VAV3 redundantly control the expression of genes encoding all the main enzymes of the mevalonate pathway in a RAC1‐ and SREBF‐dependent manner. Furthermore, by using knockdown cells for transcripts of two key enzymes of this metabolic pathway (HMGCS1 and HMGCR), we observed that the activity of this VAV‐dependent anabolic program is important for maintaining optimal primary breast tumorigenesis and effective metastasis of breast cancer cells to the lung. In the former case, we found that the depletion of endogenous HMGCS1 or HMGCR leads to defects in neoangiogenesis and, to a lesser extent, to increased levels of basal apoptosis in cancer cells within the tumor mass. Interestingly, it has been shown before that simvastatin, a well‐known inhibitor of HMGCR, blocks the angiogenic activity of mouse 4T1 and human MDA‐MB‐231 cells through an AMPK (5′ AMP‐activated protein kinase)‐ and HIF1α (hypoxia‐inducible factor 1α)‐dependent mechanism [61]. The antiangiogenic effects of simvastatin have also been observed in HER2^+^ colorectal models, although in this case the effects were associated with reduced secretion of vascular endothelial growth factor [62]. However, to the best of our knowledge, this is the first study in which such an effect is observed by direct interference with the normal levels of endogenous HMGCR or the upstream HMGCS1 in breast cancer cells. Our results also indicate that, in addition to HMGCR, the targeting of other enzymes of the mevalonate pathway (e.g., HMGCS1) could be an option for impairing the fitness of breast cancer tumors through the reduction of neoangiogenesis and the increase in basal rates of cancer cell apoptosis within the tumor mass.

The activation of the mevalonate pathway by VAV2 and VAV3 is RAC1‐ and SREBF‐dependent. The lack of stimulation of SREBF activity by active versions of RHOG, RHOA, or CDC42 suggests that this pathway must be engaged independently of PAK (which is stimulated by both RAC1 and CDC42) and serum response factors (which can be stimulated by all the GTPases that have been tested in our experiments). Using chemical inhibitors for specific signaling proteins, we also found that the stimulation of SREBF activity by RAC1^Q61L^ cannot be blocked by PI3K inhibitors or with the mTORC1 inhibitor rapamycin. In contrast, it could be abrogated when using dual mTORC1 and mTORC2 inhibitors such as torin1 and torkinib or a MEK inhibitor (U0126). This suggests that this pathway might be regulated by rapamycin‐insensitive downstream targets of mTORC1 (e.g., 4E‐BP1, lipin1) [4, 54, 63]. Given the PI3K independence of this pathway, it is likely that this downstream route could be engaged by an mTORC2‐dependent mechanism that leads to the stimulation of the AKT–mTORC1 axis. In line with this latter possibility, we have found that torin1 and torkinib, but not rapamycin, also inhibit the basal phosphorylation of the mTORC2‐targeted Ser^473^ residue of AKT in 4T1 cells (Fig. S2D, top panel). Previous reports have shown that mTORC2–AKT‐dependent mechanisms for SREBF activation are present in glioblastoma and liver [64, 65]. In addition, it has been shown that the MEK–ERK axis can contribute to SERBF activation through a phosphorylation‐mediated mechanism [52, 53].

It is likely that the effect of VAV2 and VAV3 on the mevalonate pathway could result in a widespread generation of the anabolic products of this route (Fig. 1B), although this awaits confirmation using detailed metabolic tracing experiments in KD_2/3_ cells. Despite this, our data demonstrated that the stimulation of the mevalonate pathway by VAV2 and VAV3 is clearly associated with the promotion of protumorigenic programs. However, it is important to underscore that the connection between mevalonate pathway activity and protumorigenic programs does not always take place. For example, a recent report has shown that the overexpression of the scaffolding protein p140^CAS^ (p130 Crk‐associated substrate‐associated protein of 140 kDa) can induce the mevalonate pathway while having an overall tumor suppressor effect in breast cancer cells [66]. This is because the overexpressed p140^CAS^ protein promotes the activation of three processes that counterbalance the effect on the mevalonate pathway: (a) exportation of the generated cholesterol to the extracellular space via the stimulation of ABC (ATP‐binding cassette) transporters; (b) transfer of the generated cholesterol to the plasma membrane, which leads to increased membrane rigidity and decreased cell motility of breast cancer cells overexpressing p140^CAS^; and (c) reduced incorporation of RAC1 into lipid rafts that, in turn, precludes optimal RAC1 signaling in p140^CAS^‐expressing cells [66]. So, in this case, the overexpression of p140^CAP^ seems to be detrimental rather than beneficial for the tumorigenic activity of breast cancer cells despite its positive effect on increasing the activity of the mevalonate pathway.

Our experiments also revealed that the contributions of endogenous HMGCS1 and HMGCR to the metastatic efficiency of 4T1 cells are due to two independent causes. In the case of the depletion of endogenous *Hmgcs1*, we observed that the lack of metastatic development was likely a mere indirect effect of the reduced tumor masses formed by the knockdown cells in the recipient mice. The same probably applies to *Hmgcr* knockdown cells, given that these cells can form tumor masses similar to those found in the recipient mice transplanted with the *Hmgcs1* knockdown cells. However, in this case, the *Hmgcr* knockdown cells also exhibited metastasis‐specific defects associated with the extravasation efficiency and subsequent fitness of the breast cancer cells within the lung niche. Similarly to the *in vivo* experiments, we have observed that *Hmgcr* knockdown cells have more aggravated defects in cell culture than the *Hmgcs1* counterparts when tested in MTT metabolization, ferroptosis, and mitochondrial metabolism assays. The reason for the more penetrant effect of the *Hmgcr* knockdown is unknown. It is possible that the depletion of HMGCR could induce a major metabolic impact on cells for two reasons. On the one hand, its depletion must have a major effect on the biosynthetic output of the mevalonate route, given that it is the key rate‐limiting step of this metabolic route. On the other hand, it is possible that the depletion of HMGCS1 could lead to a more general compensatory effect on the expression of other downstream enzymes of the same pathway, including HMGCR itself as shown in our qRT–PCR experiments (Fig. S3B,C). Alternatively, it is also feasible that HMGCS1 and HMGCR may have functions outside their canonical involvement in the mevalonate pathway. This idea is supported by previous studies showing that knocking down the *MVK* transcript, but not the *PMVK* or the *MVD* mRNAs, triggers the degradation of mutant TP53 in several cancer cell lines [67]. Further studies are needed to clarify this issue.

The observation indicating that the mevalonate pathway‐encoding genes are poorly expressed in nonmetastatic cell lines further indicates that this metabolic pathway is likely associated with the metastatic proficiency of breast cancer cells. Our functional assays also indicated that the knockdown of endogenous VAV2 and VAV3 proteins had a more widespread functional impact on the tumorigenic and metastatic properties of 4T1 cells than the depletion of either HMGCR or HMGCS1 (Fig. S8). Thus, in a previous work, we showed using orthotopic transplant experiments that *Vav2*;*Vav3* knockdown 4T1 cells exhibit proliferative, survival, angiogenic, and metastatic defects [27]. Elimination of some of the distal transcriptional targets of the VAV‐dependent pathway characterized in that study also elicited a greater functional impact on breast tumorigenesis and lung metastasis (e.g., INHβA, TACSTD2, COX2) (Fig. S8) [27]. In contrast, the effects of knocking down endogenous *Hmgcs1* mRNA and, to a greater extent, the *Hmgcr* transcript were more limited (Fig. S8).

To further address the impact of HMGCR function on 4T1 cells, we performed genome‐wide expression analyses to identify genes whose expression was directly dependent on the expression level of this enzyme. These studies indicated that the expression of this enzyme is important for sustaining the expression of gene sets closely linked to proliferation‐related processes, basic metabolic programs, and immune responses that probably contribute to the protumorigenic and prometastatic functions of this enzyme. We also found that the expression of this enzyme is associated with the downregulation of critical gene expression programs, such as those regulated by estrogens, Hedgehog, NOTCH, TP53, tumor necrosis factor α, or the WNT–β‐catenin axis. This latter observation is rather unexpected because the activity of those pathways (e.g., estrogens, NOTCH, Hedgehog) has been shown to be dependent on the mevalonate route in previous studies [68, 69, 70, 71]. Using immunoblot analysis, we also found a clear connection between proper levels of HMGCR and optimal PI3K signaling in insulin growth factor 1‐stimulated 4T1 cells.

We also assessed whether the expression levels of mevalonate pathway‐encoding genes and/or mevalonate pathway‐associated gene expression programs could help stratifying breast cancer patients. We did not find any statistically significant differences in breast cancer patient stratification according to the expression of either the *HMGCR* transcript or the mRNAs for the entire mevalonate pathway. In contrast, we found that the HMGCR‐dependent gene signature reported in this work has some prognostic value for breast cancer patients according to specific clinical features (distant recurrence‐free survival for nonstratified patients and for chemotherapy‐insensitive patients). This result is interesting, given that the mevalonate pathway has been associated with chemoresistance in small‐cell lung cancer and triple‐negative breast cancer [11, 72]. The stratification power of the HMGCR‐dependent gene signature was similar to (or even better) that of the other gene signatures tested in the present study (VAV2;VAV3‐dependent signature A; MammaPrint). However, this power is significantly worse than that provided by the VAV2;VAV3‐dependent signature B, which is directly associated with the ability of 4T1 cells to develop lung metastasis [27]. Indeed, this latter signature has prognostic value for all clinical parameters examined in this study, as previously shown [27, 28]. Taken together, these data suggest that the mevalonate pathway is an important, but not the unique VAV2;VAV3‐dependent pathway that contributes to the tumorigenic and metastatic properties of 4T1 cells. They also further emphasize the important role that both VAV2 and VAV3 play in breast cancer, namely the engagement of different distal transcriptional targets that contribute to overlapping but not identical manners to the malignant program of breast cancer cells. Future studies will undoubtedly unveil new VAV2;VAV3‐regulated programs involved in this tumorigenic process.

## 5 Conclusions

This study highlights the critical role of two VAV family members, VAV2 and VAV3, in regulating the mevalonate pathway in breast cancer cells through a RAC1‐ and SREBF‐dependent mechanism. Using loss‐of‐function experiments, we demonstrated that the expression of two upstream enzymes of this metabolic pathway, HMGCS1 and HMGCR, is crucial for the primary tumorigenesis of breast cancer cells *in vivo*. This process is linked to the regulation of both pro‐survival and neoangiogenic signals in the tumor mass. The depletion of HMGCR also impairs the extravasation of circulating breast cancer cells and their subsequent survival in the lung parenchyma. Consistent with these findings, *in silico* analyses have shown that the expression of mevalonate pathway‐encoding transcripts correlates with the metastatic efficiency of several breast cancer cell lines. HMGCR‐dependent gene signatures also have predicted value to stratify the long‐term survival of breast cancer patients. The impact of HMGCS1 and HMGCR in different cell parameters has been evaluated in cell culture, further indicating the more penetrant effect of HMGCR in the functional status of breast cancer cells. Collectively, these data suggest that the pharmacological inhibition of VAV proteins or mevalonate pathway enzymes, specifically HMGCR, may represent potential treatment avenues for breast cancer patients. However, they also indicate that inhibitors targeting mevalonate pathway components could lead to the compensatory upregulation of other enzymes in the same metabolic route, thus potentially reducing the efficacy of such treatments.

## Conflict of interest

The authors declare no conflict of interest.

## Author contributions

JC and IF‐P participated in all the experimental work and contributed to the artwork design and manuscript writing. LFL‐M performed *in silico* analyses, generated cell lines, analyzed the data, and contributed to the artwork design. RG‐G, BC, and PC carried out metastasis‐related experiments. XRB conceived the work, analyzed the data, wrote the manuscript, and performed the final editing of the figures.

### Peer review

The peer review history for this article is available at https://www.webofscience.com/api/gateway/wos/peer‐review/10.1002/1878‐0261.13716.

## Supporting information


**Fig. S1.** The expression of mevalonate pathway genes correlates with the metastatic potential of mouse breast cancer cell lines.
**Fig. S2.** Effect of inhibitors for the PI3K and ERK pathways in 4T1 cells.
**Fig. S3.** Characterization of *Hmgcs1* and *Hmgcr* knockdown cells in cell culture.
**Fig. S4.** Characterization of metabolic parameters in *Hmgcs1* and *Hmgcr* knockdown 4T1 cells.
**Fig. S5.** HMGCR affects gene expression programs associated with different cancer hallmarks.
**Fig. S6.** Effect of the *Hmgcr* knockdown and Hmgcr overexpression in the PI3K and ERK signaling pathways.
**Fig. S7.** HMGCR affects gene expression programs associated with different cancer hallmarks.
**Fig. S8.** Depiction of the role of VAV proteins and distal targets in mammary tumorigenesis and metastasis.

## Data Availability

All relevant data are available from the corresponding author upon reasonable request. A Materials Transfer Agreement may be required in the case of potential commercial applications. As indicated above, the microarray data generated in this study have been deposited in the GEO database (Reference number: GSE253308).
