# Supplementary material for: The mevalonate pathway contributes to breast primary tumorigenesis and lung metastasis
Source: Mol Oncol. 2024 Aug 9;19(1):56–80. doi: 10.1002/1878-0261.13716 (PMC11705731; doi:10.1002/1878-0261.13716)
Supplement: Supplementary file 1 — Fig. S1. The expression of mevalonate pathway genes correlates with the metastatic potential of mouse breast cancer cell lines. Fig. S2. Effect of inhibitors for the PI3K and ERK pathways in 4T1 cells. Fig. S3. Characterization of Hmgcs1 and Hmgcr knockdown cells in cell culture. Fig. S4. Characterization of metabolic parameters in Hmgcs1 and Hmgcr knockdown 4T1 cells. Fig. S5. HMGCR affects gene expression programs associated with different cancer hallmarks. Fig. S6. Effect of the Hmgcr knockdown and Hmgcr overexpression in the PI3K and ERK signaling pathways. Fig. S7. HMGCR affects gene expression programs associated with different cancer hallmarks. Fig. S8. Depiction of the role of VAV proteins and distal targets in mammary tumorigenesis and metastasis. [file MOL2-19-56-s001.pdf]

Supplementary Information for

THE MEVALONATE PATHWAY FAVORS BREAST PRIMARY TUMORIGENESIS AND LUNG METASTASIS

by

Javier Conde, Isabel Fernández-Pisonero *et al*.

This PDF file includes:

Supplementary Figures 1 to 7 and legends

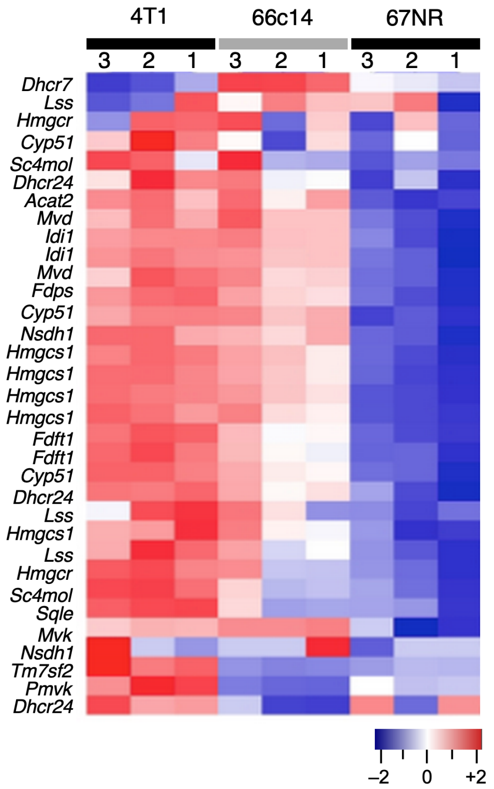

FIGURE S1. The expression of mevalonate pathway genes correlates with the metastatic potential of mouse breast cancer cell lines

Heatmap showing the expression of mevalonate pathway genes (left) in the indicated cell lines (top). Genes upregulated and downregulated are shown in red and blue color, respectively. The gradient reflects the relative fold-change variation in the expression of the interrogated genes. The replicates for each cell line are shown on the top.

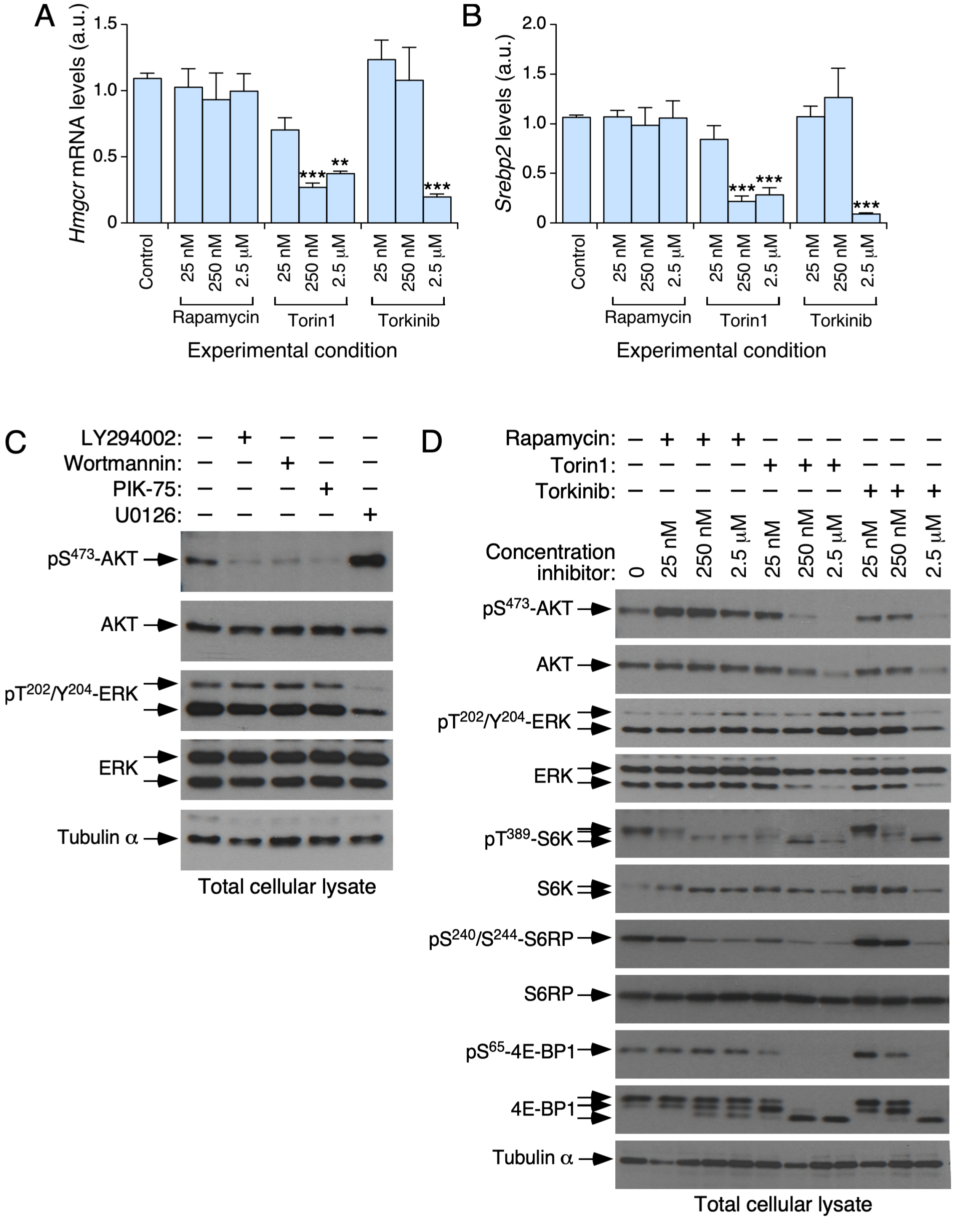

FIGURE S2. Effect of inhibitors for the PI3K and ERK pathway in 4T1 cells

(A and B) Effect of the indicated inhibitors (bottom) in the expression level of the transcripts for HMGCR (A) and SREBP2 (B) in 4T1 cells.

(C) Representative Western blot analyses showing the effect of the indicated inhibitors (top) in the phosphorylation levels of Akt and ERK in 4T1 cells. Levels of tubulin α in each sample were used as loading control (bottom panel). Similar results were obtained in two additional independent experiments.

(D) Representative Western blot analysis showing the effect of the indicated inhibitors (top) for PI3K and ERK signaling elements in 4T1 cells. Levels of tubulin α in each sample were used as loading control (bottom panel). Similar results were obtained in two additional independent experiments.

Data shown in panels A and B represent the mean ± SEM. **, $P \leq 0.01$; ***, $P \leq 0.001$ relative to the untreated control cell line (Student *t*-test, $n = 3$ independent experiments).

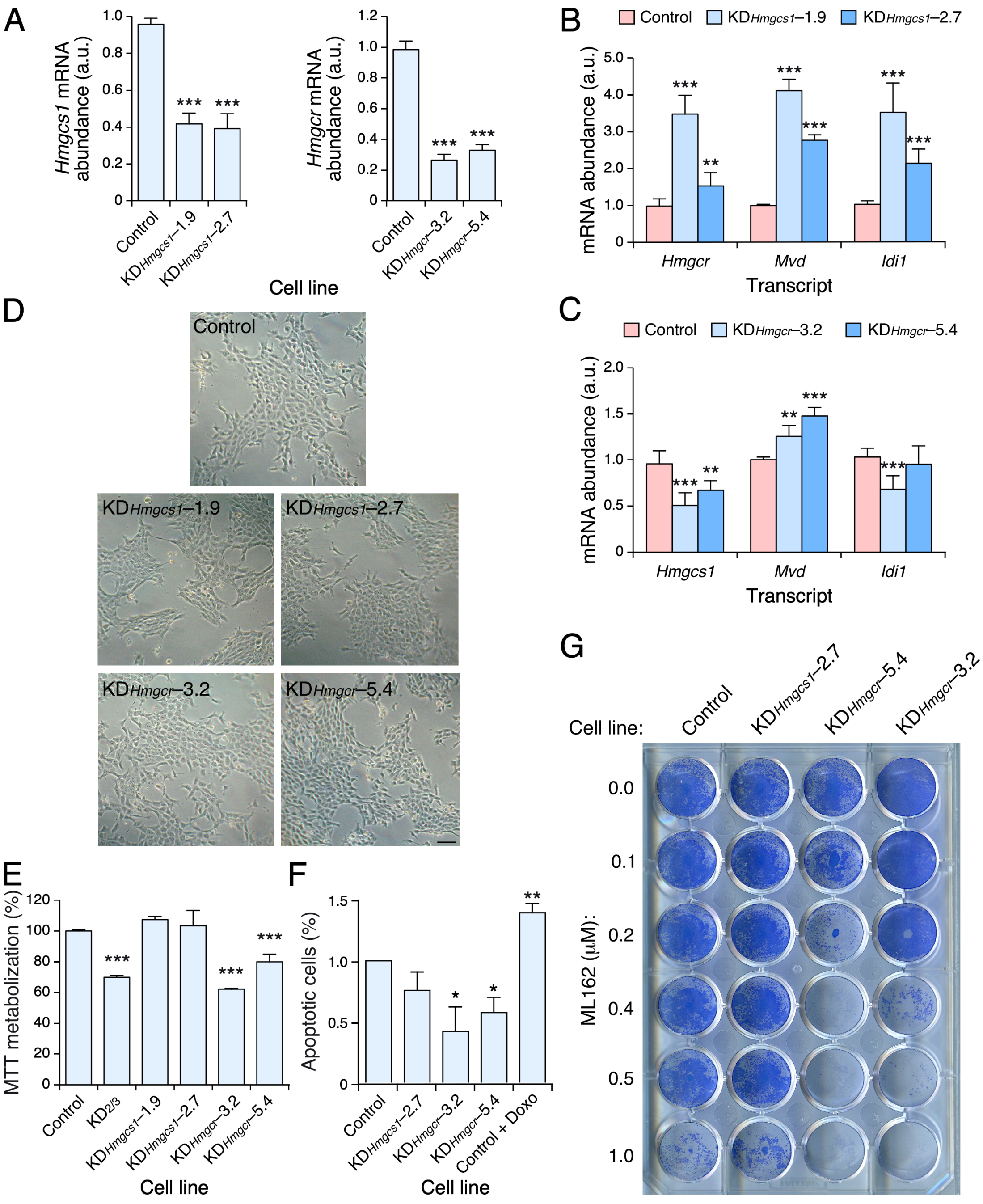

FIGURE S3. Characterization of *Hmgcs1* and *Hmgcr* knockdown cells in cell culture

(A) Abundance of *Hmgcs1* (left) and *Hmgcr* (right) transcripts in indicated 4T1 cell lines (bottom). Values are shown relative to the abundance of each transcript in the control cell line, which was given an arbitrary number of 1. a.u., arbitrary units. Control, cells expressing the empty pLKO vector.

(B and C) Levels of indicated transcripts (B and C, bottom) in the indicated 4T1 cell lines (insets). Values are shown relative to the abundance of each transcript in the control cell line, which was given an arbitrary number of 1.

(D) Representative images showing the morphology of the indicated 4T1 cell lines in 2D culture. Scale bar, 50 μm.

(E and F) MTT metabolization activity (E) and apoptotic rates (F) of indicated cells lines (bottom) using a MTT assay. Values are shown as a relative percentage of values obtained with the control cell line, which was given an arbitrary value of 100 (E) or 1 (F).

(G) Response of indicated 4T1 cell lines (top) to the ML162. Upon culturing for 24 h with the indicated concentrations of the inhibitor (left), cells were stained with Giemsa and photographed. Data are representative of 3 independent experiments.

Data shown in panels A, B, C, E and F represent the mean ± SEM. *, $P \leq 0.05$; **, $P \leq 0.01$; ***, $P \leq 0.001$ relative to the control cell line (Student *t*-test, $n = 3$ independent experiments).

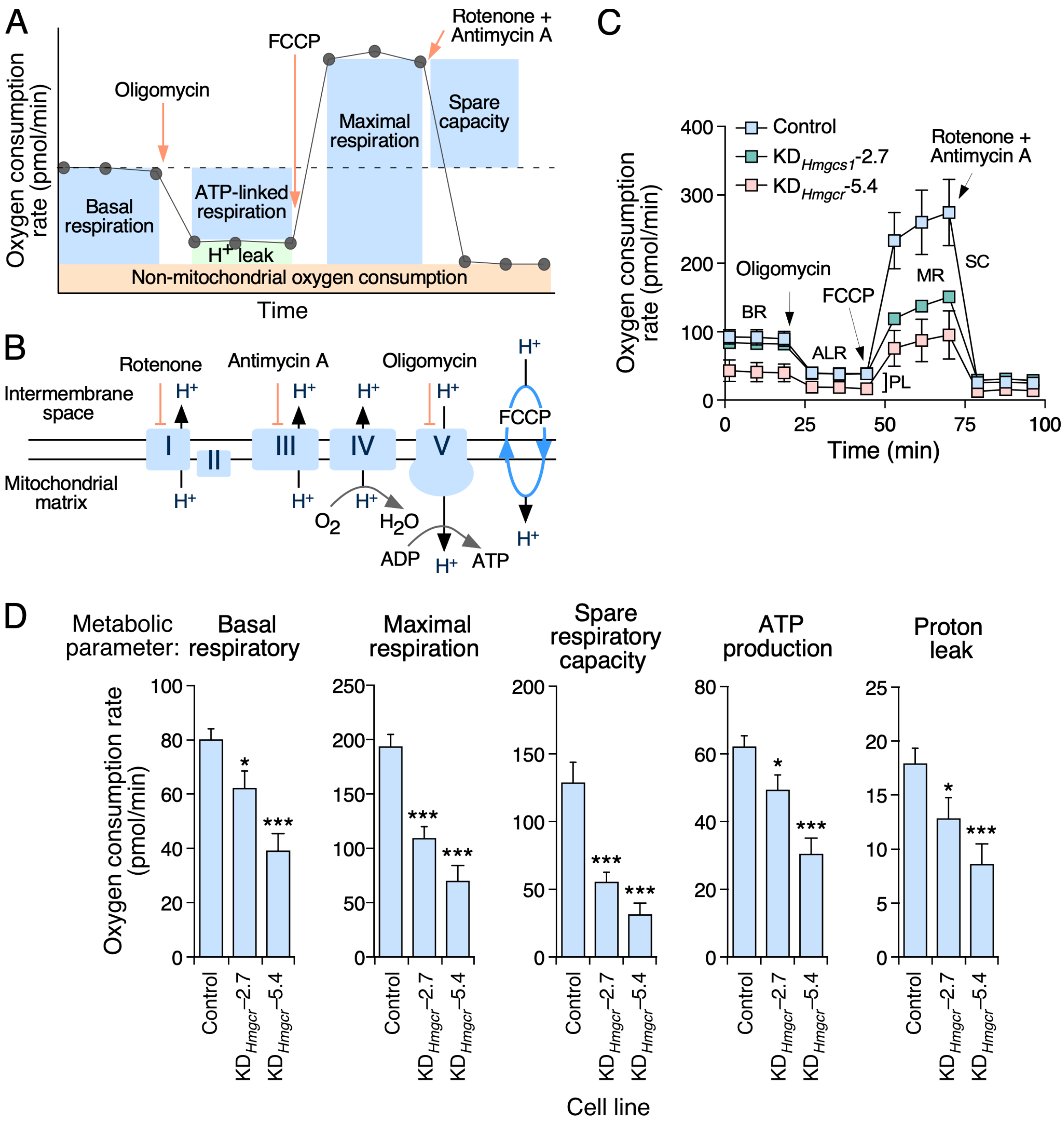

FIGURE S4. Characterization of metabolic parameters in *Hmgcs1* and *Hmgcr* knockdown 4T1 cells

(A) Schematic representation of the expected respiratory profile obtained using the Seahorse Mito Stress test. Metabolic parameters (blue boxes) were determined based on the measurement of oxygen consumption rates following addition of the indicated drugs at the specified time points. FCCP, phenylhydrazone.

(B) Depiction of the electron transport chain. I to V represent the complexes participating in the oxidative phosphorylation process. Inhibitors and targets used in the Seahorse tests are indicated.

(C) Representative example of oxygen consumption rates in indicated 4T1 cells. BR, basal respiration; ALR, ATP-linked respiration; PL, proton leak; MR, maximal respiration; SC, spare capacity.

(D) Quantitation of indicated metabolic parameters (top) in the 4T1 cell lines shown in the bottom. Bars represent the mean ± SEM. *, $P \leq 0.05$; ** $P \leq 0.01$; ***, $P \leq 0.001$ (Student *t*-test*, n* = 4 independent experiments).

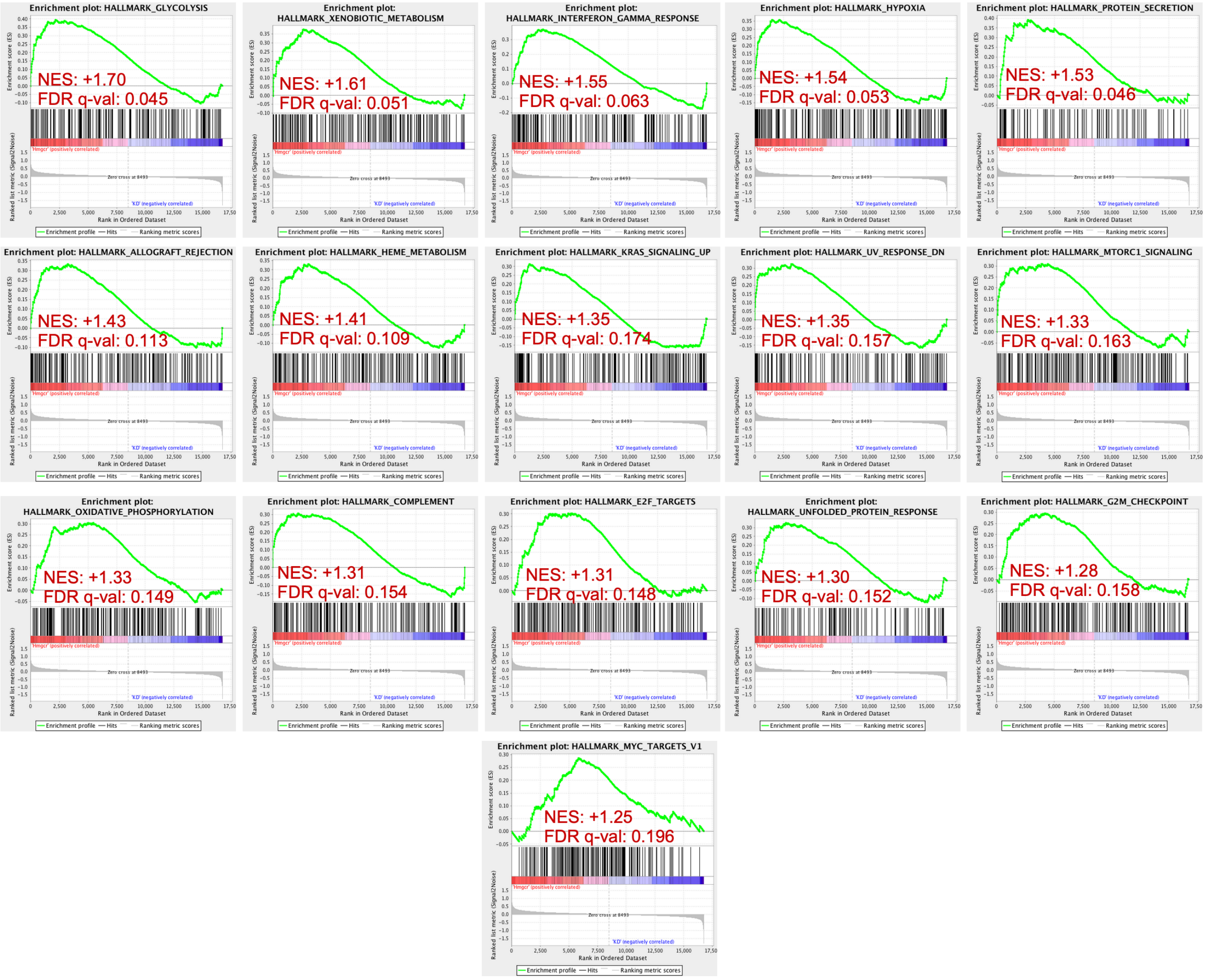

FIGURE S5. HMGCR affects gene expression programs associated with different cancer hallmarks

GSEAs showing the main gene signatures enriched in the up-regulated subset of the HMGCR-dependent transcriptome of 4T1 cells. The normalized enrichment score (NES) and false discovery rate values (FDR *q* value) are indicated inside each GSEA graph.

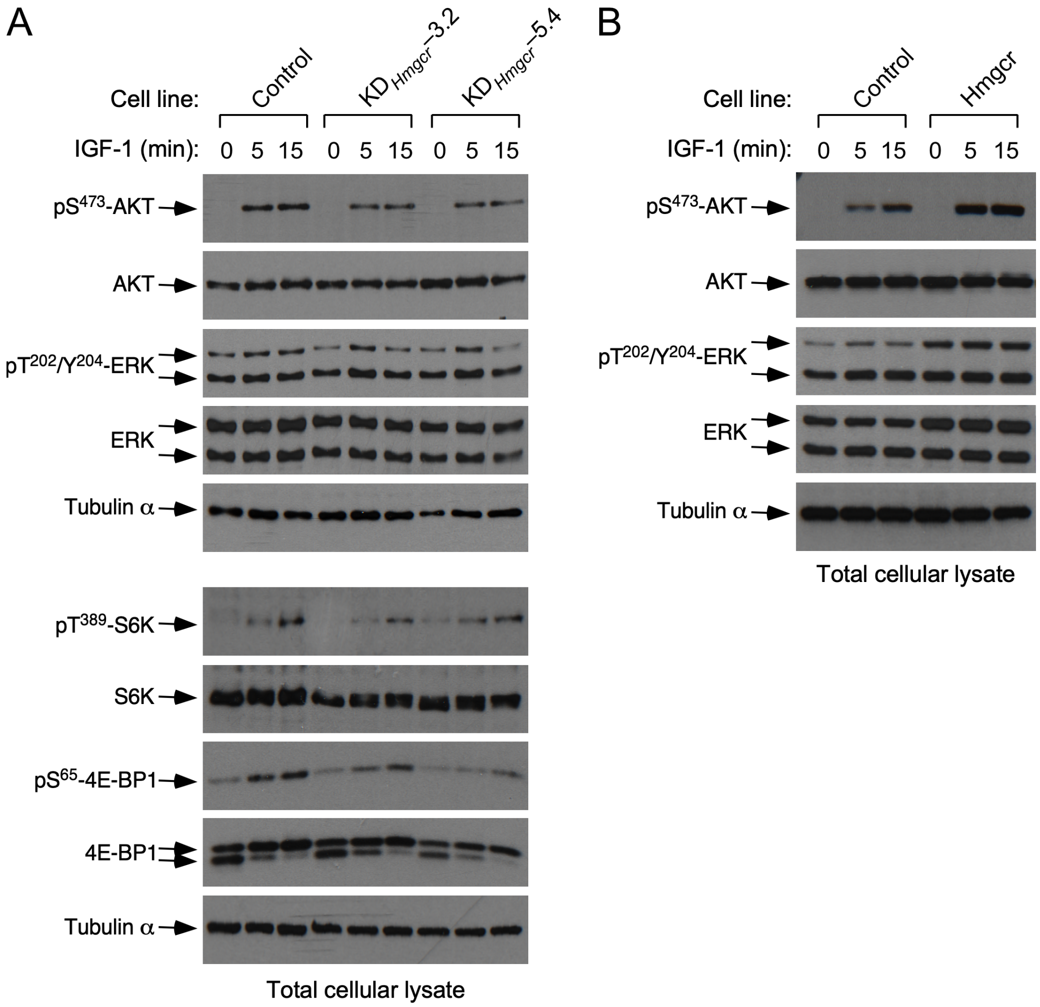

FIGURE S6. Effect of the *Hmgcr* knockdown and Hmgcr overexpression in the PI3K and ERK signaling pathways

(A) Representative Western blot analyses showing either the phosphorylation or total levels of the indicated signaling proteins in IGF-1 stimulated control and *Hmgcr* knockdown 4T1 cells. Levels of tubulin α in each sample were used as loading control (fifth and tenth panels from top). Similar results were obtained in two additional independent experiments. Control, cells containing the pLKO vector alone; IGF-1, insulin growth factor 1.

(B) Representative Western blot analyses showing either the phosphorylation or total levels of the indicated signaling proteins in IGF-1 stimulated control and Hmgcr overexpressing 4T1 cells. Levels of tubulin α in each sample were used as loading control (bottom panel). Similar results were obtained in three additional independent experiments. Control, cells containing the empty pLVX vector.

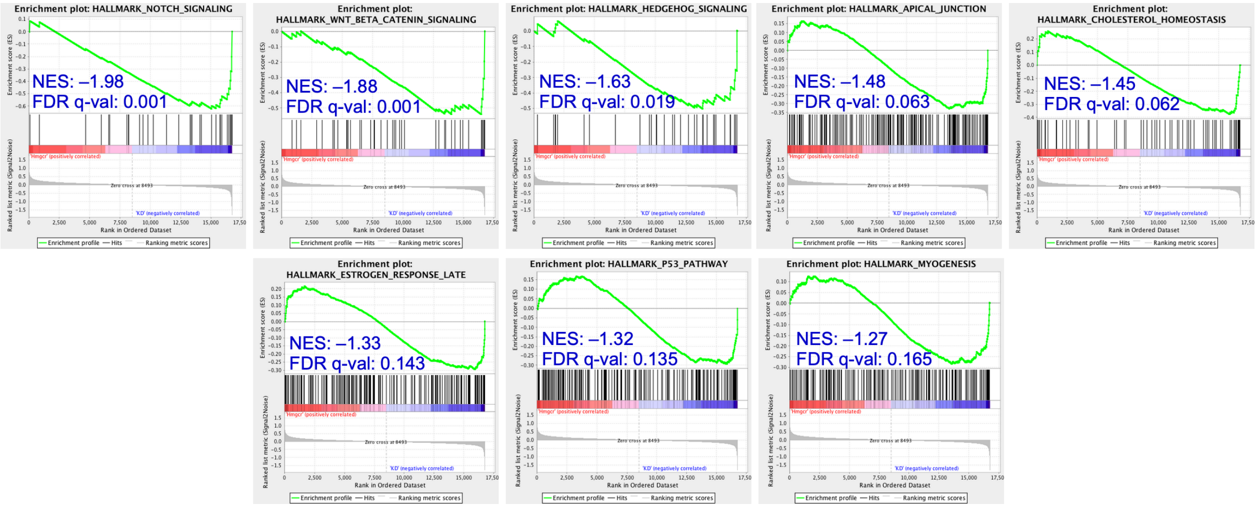

FIGURE S7. HMGCR affects gene expression programs associated with different cancer hallmarks

GSEAs showing the main gene signatures enriched in the down-regulated subset of the HMGCR-dependent transcriptome of 4T1 cells. The normalized enrichment score (NES) and false discovery rate values (FDR q value) are indicated inside each GSEA graph.

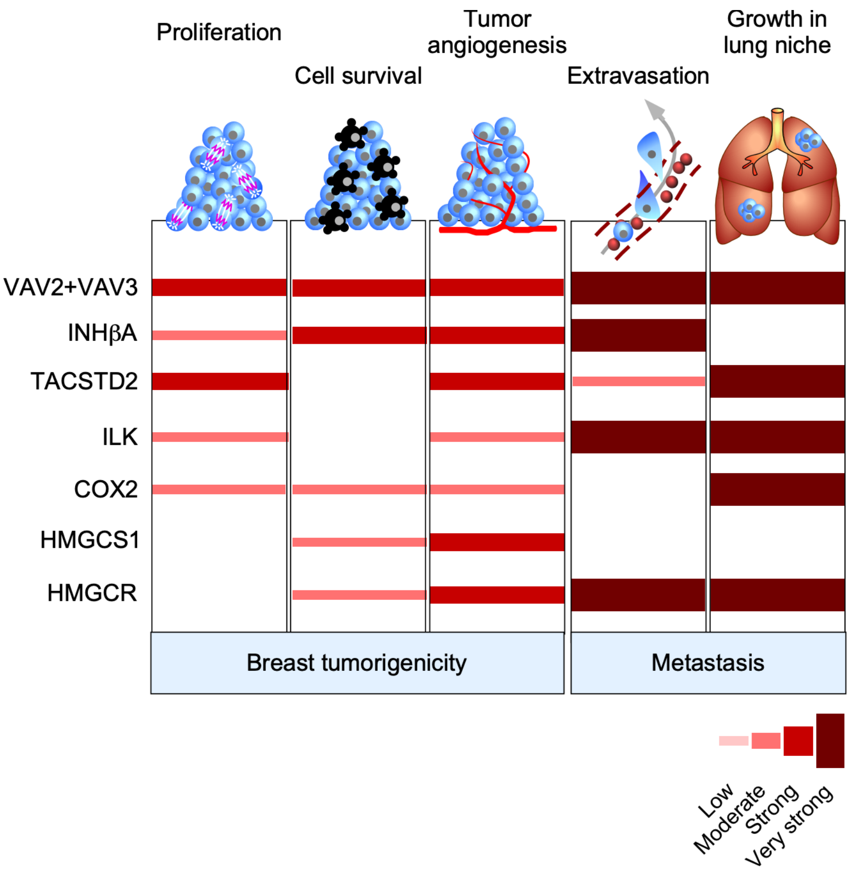

FIGURE S8. Depiction of the role of Vav proteins and distal targets in mammary tumorigenesis and metastasis

This figure integrates data from a previous publication (Citterio et al., *Sci Signal* 2022, PMID: 23033540) and the results obtained in the present work (HMGCS1, HMGCR). The impact of the depletion of an interrogated protein (left) on the indicated process (top) is proportional to the thickness and darkness of the horizontal bars (see scale at the bottom).
